# Development of a Machine Learning–Based Predictive Model for Postoperative Delirium in Older Adult Intensive Care Unit Patients: Retrospective Study

**DOI:** 10.2196/67258

**Published:** 2025-06-19

**Authors:** Houfeng Li, Qinglai Zang, Qi Li, Yanchen Lin, Jintao Duan, Jing Huang, Huixiu Hu, Ying Zhang, Dengyun Xia, Miao Zhou

**Affiliations:** 1 Graduate School Hebei North University Zhangjiakou China; 2 School of Anesthesiology Naval Medical University Shanghai China; 3 Information Center The Second Affiliated Hospital of Naval Medical University Shanghai China; 4 Department of Anesthesiology Shanghai Ninth People’s Hospital Shanghai Jiao Tong University School of Medicine Shanghai China; 5 School of Health Science and Engineering University of Shanghai for Science and Technology Shanghai China; 6 Graduate School Wannan Medical College Wuhu China; 7 Department of Anesthesiology The First Affiliated Hospital of Hebei North University Zhangjiakou China; 8 Department of Anesthesiology The Affiliated Cancer Hospital of Nanjing Medical University, Jiangsu Cancer Hospital, Jiangsu Institute of Cancer Research Nanjing Medical University Nanjing China

**Keywords:** older adults, delirium, machine learning, artificial intelligence, delirium assessment, predictive modeling, intensive care, XGB, extreme gradient boosting

## Abstract

**Background:**

Delirium is a prevalent phenomenon among patients admitted to the geriatric intensive care unit (ICU) and can adversely impact prognosis and augment the risk of complications.

**Objective:**

We aimed to construct and validate a predictive model for postoperative delirium state in older ICU patients, providing timely and effective early identification of high-risk individuals and assisting clinicians in decision-making.

**Methods:**

The data from patients admitted to the ICU for over 24 hours were extracted from the Medical Information Marketplace for Intensive Care IV (MIMIC-IV) database and the eICU Collaborative Research Database (eICU-CRD). The MIMIC-IV data were split (7:3) into training and internal validation sets, while the eICU-CRD data served as an external validation set. Delirium predictions were conducted for the subsequent prediction windows (12 h, 24 h, 48 h, and whole stay time) using data from the first 24 hours post admission. The corresponding feature variables were subjected to Boruta feature selection, and the prediction models were constructed using logistic regression, support vector classifier, random forest classifier, and extreme gradient boosting (XGB). Subsequently, model performance was evaluated using areas under the receiver operating characteristic curves (AUCs), Brier scores, and decision curve analysis, and external validation.

**Results:**

The MIMIC-IV and eICU-CRD datasets comprised 6129 and 709 patients, respectively, who were included in the analysis. Fifty-four features were selected to construct the predictive model. Regarding internal validation, the XGB model demonstrated the most effective prediction of delirium across different prediction windows. The AUCs for the 4 prediction windows (12 h, 24 h, 48 h, and whole stay time) were 0.848 (95% CI 0.826-0.869), 0.852 (95% CI 0.831-0.872), 0.851 (95% CI 0.831-0.871), and 0.844 (95% CI 0.823-0.863), respectively, and those of the external validation set were 0.777 (95% CI 0.726-0.825), 0.761 (95% CI 0.710-0.808), 0.753 (95% CI 0.704-0.798), and 0.737 (95% CI 0.695-0.777), respectively. Furthermore, the XGB model demonstrated the most accurate calibration across all prediction windows, with values of 0.129, 0.136, 0.144, and 0.148, respectively. Additionally, decision curve analysis revealed that the XGB model outperformed the other models in terms of net gain for the majority of threshold probability values. The 6 most significant predictive features identified were the first day’s delirium assessment results, type of first care unit, minimum Glasgow Coma Scale (GCS) score, Acute Physiology Score III, acetaminophen, and nonsteroidal anti-inflammatory drugs.

**Conclusions:**

The high-performance XGB model for predicting postoperative delirium state in older adult ICU patients has been successfully developed and validated. The model predicts the delirium state at 12 h, 24 h, 48 h, and whole stay time after the first day of hospitalization within the ICU. This enables physicians to identify high-risk patients early, thus facilitating the optimization of personalized management strategies and care plans.

## Introduction

Delirium is a common postoperative complication in older adult patients, characterized as an acute neuropsychiatric syndrome involving impairments in cognition, consciousness, attention, and psychomotor activity [1,2]. This condition not only significantly prolongs hospital stays and increases health care costs but is also closely associated with an increased incidence of postoperative complications and elevated mortality risk [3-7]. Of all hospital departments, intensive care units (ICUs) have the highest incidence of delirium, with rates of up to 80% in ICUs compared to 14%-29% in non-ICU inpatients [8]. This disparity may be closely associated with the severe physiological disturbances and polypharmacy commonly present in ICU patients [9,10]. Additionally, in older adult patients, the reduced ability to metabolize systemic anesthetics postoperatively leads to drug accumulation, which is significantly associated with an increased risk of ICU delirium [11,12]. Fortunately, approximately 30%-40% of cases can demonstrate significant improvement through preventive intervention [13-15]. It is therefore imperative that delirium is predicted at the earliest opportunity upon admission to the ICU, particularly in older adult patients.

Machine learning is an artificial intelligence technique that can process a substantial number of variables in a nonlinear and highly interactive manner [16]. This technology autonomously uncovers inherent patterns within data to construct adaptive models, thereby enabling computer systems to achieve intelligent decision-making and accurate predictions without explicit programming instructions [17,18]. Machine learning has been applied in the medical field for diagnosing diseases, recognizing medical images, providing treatment strategies, and predicting outcomes [19]. To date, multiple studies have developed risk prediction models for postoperative delirium in older adult patients primarily based on preoperative and intraoperative parameters [20-23]. Compared with traditional assessment methods relying on clinical symptoms, these models demonstrate superior predictive performance. However, it is noteworthy that existing studies suffer from limitations such as restricted sample sizes or lack of external validation. Their clinical applicability and research quality still require further validation through multicenter, large-sample cohort studies.

In this study, we developed machine learning models to predict subsequent delirium status in older adult postoperative patients based on first-day characteristic data after ICU admission, comprehensively collected from the Medical Information Marketplace for Intensive Care IV (MIMIC-IV) database and the eICU Collaborative Research Database (eICU-CRD). Unlike previous studies, this research innovatively established a prediction model based on real clinical scenarios. By retaining subjects with positive delirium assessment records during the baseline period and incorporating delirium evaluation results monitored within 24 h of ICU admission as model features, the study effectively addresses the modeling limitations of traditional research regarding delirium state transitions (including complex clinical processes such as initial onset, recurrence, prolongation, and remission). In addition, the study adopted a multi-time window prediction framework to systematically evaluate the model's predictive performance for both short-term and long-term outcomes, as well as variations in delirium status. This study aims to provide evidence-based support to identify precise intervention windows and optimize personalized treatment plans for postoperative delirium, with the ultimate goal of improving patient outcomes.

## Methods

### Ethical Considerations

The MIMIC-IV database and the eICU-CRD are publicly accessible and have been approved for use by the institutional review boards of Beth Israel Deaconess Medical Centre and Massachusetts Institute of Technology, in accordance with the Declaration of Helsinki. Both databases received waivers for informed consent due to the deidentification of all protected health information. After completing the Collaborative Institutional Training Initiative Program's “Data or Specimens Only Research” course (certification number: 50199917), we had the access to extract data from both the MIMIC-IV database and the eICU-CRD. Our study followed the Transparent Reporting of Multivariate Predictive Models for Individual Prognosis or Diagnosis statement [24].

### Study Population

**Figure 1:**
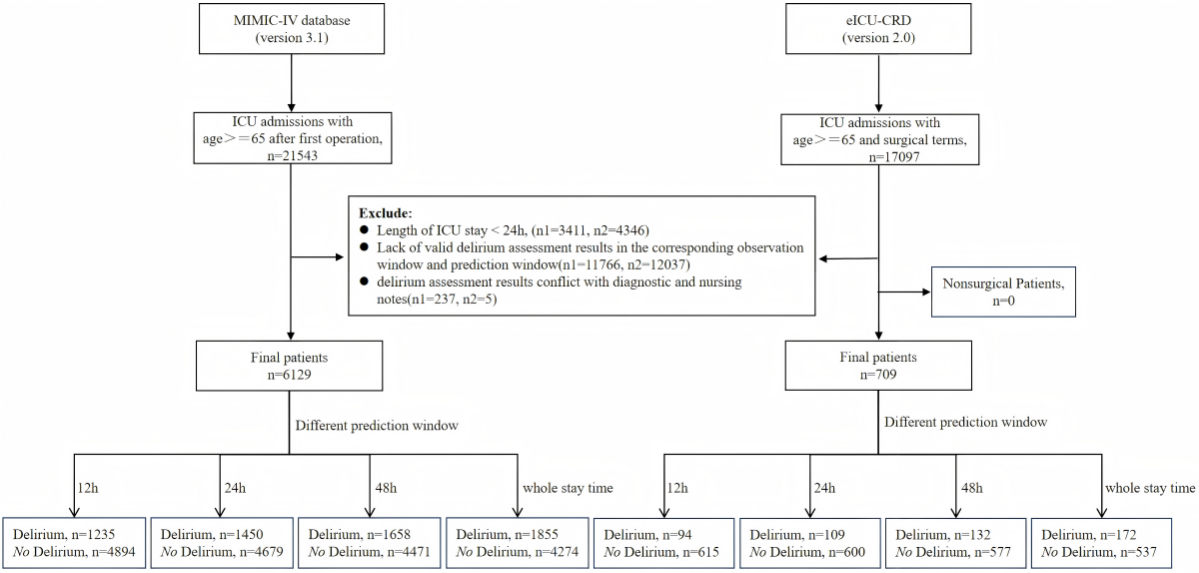
Cohort selection schema. MIMIC-IV: Medical Information Marketplace for Intensive Care IV; eICU-CRD: eICU Collaborative Research Database; ICU: intensive care unit.

The MIMIC-IV database comprises electronic health record data for 76,943 ICU admissions at Beth Israel Deaconess Medical Centre between 2008 and 2019 [25]. The eICU-CRD is a multicenter telemedicine database comprising data on over 200,000 patients admitted to 335 ICUs at 208 hospitals across the United States between 2014 and 2015 [26]. The study population comprised two cohorts: (1) patients aged ≥65 years with initial postoperative ICU admissions from the MIMIC-IV database, and (2) patients aged ≥65 years with surgery-related diagnoses in the eICU-CRD. To comprehensively and accurately include postoperative patients, we linked the surgical records from the procedures_icd table in the MIMIC database to each patient's first ICU admission record following their initial surgery. Since only the surgical date (without specific time stamps) was available and considering some surgeries might extend into the next day, we ultimately retained patients admitted to the ICU on both the day of the surgery and the following day to ensure clinical relevance between ICU data and surgical procedures. The inclusion criteria cover over 85% of patients admitted to the ICU for the first time postoperatively. In the eICU database, we included all the patient diagnosis records containing surgery-related entries, because a large number of postoperative patients did not have the corresponding postoperative diagnosis codes. Patients were excluded based on the following criteria: (1) ICU length of stay <24 hours; (2) absence of valid delirium assessments in the corresponding observation and prediction windows; and (3) delirium assessment results in contradiction to diagnosis records and nursing notes. For eICU-CRD data, after the initial screening, we further cleaned the nonsurgical patients in the limited data. No such patients were found in the data of this study. In our study, we retained patients who experienced delirium on the first day of ICU admission to assess their prognosis or recurrence risk, with no restrictions imposed on prior history of delirium before ICU admission. In total, 6129 and 709 patients from the 2 databases were included in this study. The detailed flowchart illustrating the inclusion and exclusion criteria is presented in Figure 1.

### Delirium Assessment

The primary outcome variable of this study was delirium, defined as a positive Confusion Assessment Method for the ICU screen or a score of 4 or more on the Intensive Care Delirium Screening Checklist, without any contradictions from diagnostic code information or nursing note information. The observation window in the study refers to the time period during which patient data were collected and models were derived. The prediction window in the study refers to the time period between the end of the observation window and the artificially set deadline. Specifically, we designated the first day following ICU admission as the observation window to predict delirium incidence during 4 distinct prediction windows: the subsequent 12 h, 24 h, 48 h, and whole ICU stay time. Delirium was diagnosed if at least 1 delirium assessment was positive in different prediction windows.

### Data Extraction and Processing

In light of the existing literature on delirium prediction, the availability of data in relevant databases, the ease of data extraction and monitoring in a clinical setting, the total of 54 categorical or numerical variables were identified that met the aforementioned criteria and were subsequently categorized into the following domains: demographic data, vital signs, laboratory values, scores, comorbidities, and treatment measures. Furthermore, the first 24 hours of delirium assessment results and the first care unit type was documented in this study, thus providing the initial overall picture of the patient's condition. As the admission diagnosis was not consistent across the application dataset, similarly, downstream variables such as outcome were not available in real time and were therefore all excluded from the study. The first 24 hours of data were extracted for the valid variables, and in addition, where patients underwent multiple vital sign measurements or laboratory tests on the first day of admission, averages were calculated and extracted to ensure reliability for subsequent analyses. A list of all the variables used can be found in Textbox 1.

Furthermore, to minimize the impact of missing data on the results, variables with more than 15% missing values were excluded from the final cohort (eg, height was excluded due to its 34.64% missing rate in our MIMIC-IV dataset). Since patient ages older than 90 years in the eICU database were masked to protect privacy, we imputed ages for these patients within the 90-100 range based on the age distribution trend of patients aged 80-89 years in the database. Missing values for other features were addressed using multiple imputation [27]. Additionally, the data were shuffled to adjust the order of the samples.

**Variables included in the prediction models.**

**Demographic data**

- Age, gender, race, and weight

**Vital signs**

- Heart rate, systolic blood pressure, diastolic blood pressure (DBP), mean blood pressure (MBP), temperature, respiratory rate, and oxygen saturation

**Laboratory results**

- Hematocrit, hemoglobin, platelets, white blood cell, anion gap, calcium, blood urea nitrogen, chloride, creatinine, glucose, sodium, potassium, international normalized ratio, prothrombin time, partial thromboplastin time, and urine output

**Comorbidity**

- Hypertension, diabetes, congestive heart failure, chronic liver disease, chronic renal disease, chronic pulmonary disease, peptic ulcer disease, tumor, and dementia

**Score**

- Sequential Organ Failure Assessment (SOFA), Glasgow Coma Scale (GCS), and Acute Physiology Score III (APSIII)

**Treatment measures**

- Invasive ventilation, renal replacement therapy, acetaminophen, anticholinergics, anticoagulants, antihistamines, antipsychotics, benzodiazepines, diuretics, general anesthetics, nonsteroidal antiinflammatory drugs, opioids, and vasopressors

**Other information**

- First 24 h delirium assessment, first care unit type

### Data Balancing

The extracted data exhibited a substantial class imbalance, with delirium-negative cases being significantly outnumbered by positive instances, leading the learning model to demonstrate a bias toward the negative class. To address this, we applied the Synthetic Minority Over-sampling Technique to generate synthetic positive samples, effectively balancing the class distribution and improving model generalizability [28].

### Feature Selection

We initially performed multicollinearity analysis using variance inflation factor, identifying multiple highly collinear feature groups (eg, international normalized ratio vs prothrombin time, mean blood pressure vs diastolic blood pressure, and hematocrit vs hemoglobin). Subsequently, feature importance evaluation was conducted through a random forest classifier (RFC) tree-based model, which enabled the removal of less important features within each collinear group.

To further refine the feature set, we implemented the Boruta feature selection algorithm [29]. The Boruta algorithm identifies the most salient features by comparing the Z-value of each feature with that of the “shadow feature.” The Z-value of each attribute is obtained from the random forest model at each iteration by copying all the real features and shuffling them sequentially. In contrast, the Z-value of the shadow is generated by randomly shuffling the real features. In multiple independent trials, if the Z-value of a real feature exceeds the maximum Z-value of a shaded feature, the real feature is deemed to be “important.” In this process, the random forest model is trained several times on the dataset, and the most important features are selected to predict the target variable. This approach helps to ensure that the strongest predictive feature factors are retained while maintaining the performance of the model.

### Parameter Tuning and Model Development

The MIMIC-IV dataset was divided into a training set and a test set, with the former accounting for 70% and the latter for 30% of the total data. The eICU-CRD dataset was used as an external validation set. In total, 4 algorithms, namely logistic regression (LR), support vector classifier, RFC and extreme gradient boosting (XGB), were used to develop the prediction model for delirium. Through Bayesian optimization, the optimal combination of hyper-parameters for LR, SVR, RFC, and XGB was automatically identified and incorporated into the corresponding model, which was then trained to achieve a high level of prediction performance.

### Model Performance Evaluation

In order to facilitate the prediction of results, auxiliary functions were created and Programming Language Theory library functions were used for the generation of receiver operating characteristic curves and confusion matrix plots. The performance of the various models was then compared using the area under the curve (AUC) values, accuracy, precision, sensitivity and specificity.

Brier Score is a performance metric primarily used to assess the accuracy and calibration of probabilistic models in binary classification tasks [30]. It measures the mean squared error between the predicted probabilities and the actual outcomes, with smaller values indicating more accurate predictions. A perfect score of 0 indicates ideal calibration where predictions exactly match reality, while higher scores (closer to the maximum value of 1) correspond to poorer performance. A decision curve is a tool used to assess the performance of a predictive model under different thresholds, and it enables users to comprehend the impact of using the model in diverse decision-making scenarios by plotting the model’s prediction curves under varying decision thresholds [31,32]. Consequently, this study uses Brier scores to evaluate the model's reliability. Decision curve analysis was used to assess the net clinical benefit. Shapley Additive Explanations (SHAP) was used to investigate the interpretability of the final predictive model.

Finally, in order to assess the generalization ability of the model and the ability of the model to predict new samples, the applicability performance of the model predictions was assessed using external validation.

### Statistical Analyses

Stata (version 17.0; StataCorp LLC), IBM SPSS Statistics (version 27.0.1; IBM Corp) and Python (version 3.9; Python Software Foundation) were applied for data processing, statistical analysis, and the development and validation of machine learning algorithms. Categorical variables were expressed as frequency and percentage and were compared using the chi-square test. Continuous variables were presented as median and interquartile distance. To compare the differences within the groups, the 2-tailed *t* test and Mann-Whitney U test were used for normal and nonnormal continuous variables, respectively. *P*<.05 indicates a statistically significant difference, and all tests were 2-tailed.

## Results

### Baseline Characteristics

The final study cohort comprised 6129 patients from the MIMIC-IV dataset, of whom 1855 (30.3%) were assessed as delirium during the remaining stay after the first day in the ICU. Additionally, 709 patients from the eICU-CRD database were included, of whom 172 (24.3%) were assessed as delirium during the remaining stay after the first day in the ICU. Table 1 presents the characteristics of patients who had delirium and those who did not in the prediction window of whole stay time. The characteristics of patients in other prediction windows are presented in Multimedia Appendices 1-3.

**Table 1:** Baseline characteristics of patients with and those without delirium in the prediction window of whole stay time.

Patients characteristics			MIMIC-IV^a^ cohort										eICU-CRD^b^ cohort								
			No delirium (n=4274)			Delirium (n=1855)			*P* value			No delirium (n=537)				Delirium (n=172)			*P* value		
**Demographic data**																					
	Age (years), median (IQR)				75.0 (69.0-82.0)			77.0 (71.0-84.0)			<.001				74.0 (69.0-80.0)			76.5 (71.0-82.0)			.009
	Male gender, n (%)				2404.0 (56.2)			960.0 (51.8)			<.001				266.0 (49.5)			98.0 (57)			.09
	Weight (kg), median (IQR)				78.6 (66.7- 91.6)			75.5 (63.3-89.1)			<.001				77.6 (65.6-91.0)			75.6 (64.9-91.3)			.79
	**Race, n (%)**										<.001										.27
		Black		296.0 (6.9)			179.0 (9.6)							59.0 (11)			27.0 (15.7)				
		White		3073.0 (71.9)			1174.0 (63.3)							429.0 (79.9)			130.0 (75.6)				
		Asian		106.0 (2.5)			29.0 (1.6)							2.0 (0.4)			1.0 (0.6)				
		Hispanic		77.0 (1.8)			35.0 (1.9)							23.0 (4.3)			4.0 (2.3)				
		Other or unknown		722.0 (16.9)			438.0 (23.6)							24.0 (4.5)			10.0 (5.8)				
**First care unit type, n (%)**											<.001										.11
	Cardiovascular ICU^c^			1912.0 (44.7)			400.0 (21.6)							109.0 (20.3)			41.0 (23.8)				
	Neurological ICU			423.0 (9.9)			283.0 (15.3)							82.0 (15.3)			35.0 (20.3)				
	Other ICU			1939.0 (45.4)			1172.0 (63.2)							346.0 (64.4)			96.0 (55.8)				
**First 24 h delirium assessment, n (%)**											<.001										<.001
	Negative			3640.0 (85.2)			677.0 (36.5)							500.0 (93.1)			100.0 (58.1)				
	Positive			634.0 (14.8)			1178.0 (63.5)							37.0 (6.9)			72.0 (41.9)				
**Vital signs, median (IQR)**																					
	Heart rate, beats/min				79.4 (71.1-89.0)			82.4 (73.1-94.2)			<.001				83.0 (74.4-91.4)			85.7 (77.5-97.0)			.005
	Systolic blood pressure, mm Hg				115.5 (106.8-126.6)			115.7 (106.4-127.3)			.47				118.4 (108.0-131.3)			119.2 (106.9-129.1)			.51
	Diastolic blood pressure, mm Hg				58.7 (53.0-65.6)			59.6 (53.7-66.2)			.003				60.8 (55.5-67.2)			60.6 (55.3-66.8)			.93
	Mean blood pressure, mm Hg				75.2 (69.9-82.1)			75.8 (70.4-82.8)			.02				78.0 (70.5-85.0)			76.9 (69.8-85.2)			.46
	Respiratory rate, beats/min				18.2 (16.4-20.3)			18.9 (16.9-21.4)			<.001				17.6 (15.7-19.9)			18.0 (15.7-21.1)			.20
	Temperature, °C				36.8 (36.6-37.0)			36.9 (36.7-37.2)			<.001				36.8 (36.6-37.1)			36.8 (36.6-37.1)			.46
	Oxygen saturation, %				97.1 (95.8-98.3)			97.5 (96.0-98.8)			<.001				97.3 (95.8-98.4)			97.4 (96.1-98.6)			.23
**Laboratory results, median (IQR)**																					
	Hematocrit, %				31.5 (28.0-35.5)			31.9 (27.6-36.0)			.31				31.3 (28.0-34.5)			30.0 (26.2-33.7)			.03
	Hemoglobin, g/dL				10.3 (9.1-11.7)			10.4 (8.9-11.7)			.21				10.4 (9.2-11.5)			9.9 (8.6-11.2)			.008
	Platelet, 10^9^/L				174.3 (132.3-228.0)			180.0 (133.3-243.0)			.02				187.6 (142.0-234.0)			176.0 (124.3-248.8)			.48
	White blood cell, 10^9^/L				11.1 (8.4-14.8)			11.9 (9.0-15.7)			<.001				11.7 (9.2-15.2)			12.2 (9.3-16.4)			.34
	Anion gap, mmol/L				13.0 (11.0-15.5)			14.3 (12.3-17.0)			<.001				10.5 (8.0-12.8)			11.0 (8.4-14.2)			.06
	Blood urea nitrogen, mg/dL				19.0 (14.0-29.0)			24.0 (16.5-39.3)			<.001				19.0 (13.0-27.0)			22.0 (14.8-35.8)			<.001
	Calcium, mg/dL				8.3 (8.0-8.7)			8.3 (7.9-8.7)			.91				8.2 (7.8-8.5)			8.2 (7.7-8.7)			.77
	Chloride, mmol/L				105.0 (101.3-107.5)			105.0 (101.0-108.0)			.36				104.5 (102.0-108.0)			106.0 (103.0-110.0)			<.001
	Creatinine, mg/dL				1.0 (0.8-1.4)			1.1 (0.8-1.7)			<.001				1.0 (0.7-1.4)			1.2 (0.9-1.7)			<.001
	Glucose, mg/dL				127.0 (108.7-150.0)			135.0 (111.3-170.8)			<.001				139.8 (118.0-162.0)			137.0 (117.4-159.7)			.44
	Sodium, mmol/L				138.3 (136.0-140.3)			139.0 (136.0-142.0)			<.001				138.3 (136.0-140.5)			140.0 (137.0-142.4)			<.001
	Potassium, mmol/L				4.2 (3.9-4.5)			4.2 (3.9-4.5)			.56				4.2 (3.9-4.6)			4.1 (3.8-4.5)			.23
	International normalized ratio				1.3 (1.2-1.4)			1.3 (1.2-1.5)			<.001				1.4 (1.2-1.6)			1.4 (1.2-1.7)			.15
	Prothrombin time, s				14.0 (12.5-15.4)			14.4 (12.5-16.2)			<.001				16.1 (14.3-18.3)			16.5 (13.7-19.8)			.16
	Partial thromboplastin time, s				31.2 (27.8-38.0)			31.8 (27.7-39.3)			.20				35.5 (35.4-35.6)			35.5 (35.3-35.6)			.04
	Urine output, mL				1485.0 (993.0- 2155.0)			1225.0 (745.0-1845.0)			<.001				1337.7 (875.0-1775.0)			1096.5 (650.0-1822.5)			.02
**Comorbidity, n (%)**																					
	Hypertension				3355.0 (78.5)			1482.0 (79.9)			.22				94.0 (17.5)			36.0 (20.9)			.31
	Diabetes				1390.0 (32.5)			686.0 (37)			<.001				70.0 (13)			26.0 (15.1)			.49
	Congestive heart failure				1434.0 (33.6)			750.0 (40.4)			<.001				43.0 (8)			13.0 (7.6)			.85
	Chronic renal disease				1043.0 (24.4)			575.0 (31)			<.001				39.0 (7.3)			19.0 (11)			.12
	Chronic liver disease				343.0 (8)			169.0 (9.1)			.16				6.0 (1.1)			4.0 (2.3)			.27
	Chronic pulmonary disease				1097.0 (25.7)			571.0 (30.8)			<.001				51.0 (9.5)			16.0 (9.3)			.94
	Peptic ulcer disease				123.0 (2.9)			73.0 (3.9)			.03				4.0 (0.7)			1.0 (0.6)			0.99
	Tumor				714.0 (16.7)			272.0 (14.7)			.05				112.0 (20.9)			32.0 (18.6)			.52
	Dementia				106.0 (2.5)			236.0 (12.7)			<.001				6.0 (1.1)			5.0 (2.9)			.12
**Score, median (IQR)**																					
	GCS^d^				15.0 (14.0-15.0)			14.0 (12.0-15.0)			<.001				14.0 (11.0-15.0)			13.0 (8.0-14.0)			<.001
	SOFA^e^				4.0 (2.0-6.0)			6.0 (3.0-8.0)			<.001				5.0 (4.0-7.0)			7.0 (5.0-9.0)			<.001
	APSIII^f^				38.0 (30.0-48.0)			49.0 (38.0-63.0)			<.001				40.0 (30.0-52.0)			49.5 (35.0-69.5)			<.001
**Treatment measures, n (%)**																					
	Renal replacement therapy				130.0 (3)			100.0 (5.4)			<.001				18.0 (3.4)			6.0 (3.5)			.93
	Invasive ventilation				1695.0 (39.7)			1190.0 (64.2)			<.001				199.0 (37.1)			82.0 (47.7)			.01
	Acetaminophen				3338.0 (78.1)			1224.0 (66)			<.001				270.0 (50.3)			100.0 (58.1)			.07
	Anticholinergics				1332.0 (31.2)			582.0 (31.4)			.87				33.0 (6.1)			22.0 (12.8)			.005
	Anticoagulants				2759.0 (64.6)			1327.0 (71.5)			<.001				197.0 (36.7)			59.0 (34.3)			.57
	Antihistamines				234.0 (5.5)			70.0 (3.8)			.005				34.0 (6.3)			8.0 (4.7)			.42
	Antipsychotics				157.0 (3.7)			204.0 (11)			<.001				6.0 (1.1)			5.0 (2.9)			.15
	Benzodiazepines				650.0 (15.2)			290.0 (15.6)			.67				87.0 (16.2)			36.0 (20.9)			.15
	Diuretics				1739.0 (40.7)			713.0 (38.4)			.10				130.0 (24.2)			42.0 (24.4)			.96
	General anesthetics				1576.0 (36.9)			1021.0 (55)			<.001				68.0 (12.7)			32.0 (18.6)			.05
	NSAIDs^g^				2259.0 (52.9)			692.0 (37.3)			<.001				105.0 (19.6)			43.0 (25)			.13
	Opioids				3672.0 (85.9)			1645.0 (88.7)			.003				276.0 (51.4)			99.0 (57.6)			.16
	Vasopressors				2087.0 (48.8)			1004.0 (54.1)			<.001				87.0 (16.2)			48.0 (27.9)			<.001

^a^MIMIC-IV: Medical Information Marketplace for Intensive Care IV.

^b^eICU-CRD: eICU Collaborative Research Database.

^c^ICU: intensive care unit.

^d^GCS: Glasgow Coma Scale.

^e^SOFA: Sequential Organ Failure Assessment.

^f^APSIII: Acute Physiology Score III.

^g^NSAIDs: nonsteroidal antiinflammatory drugs.

### Evaluation of Model Performance

**Figure 2:**
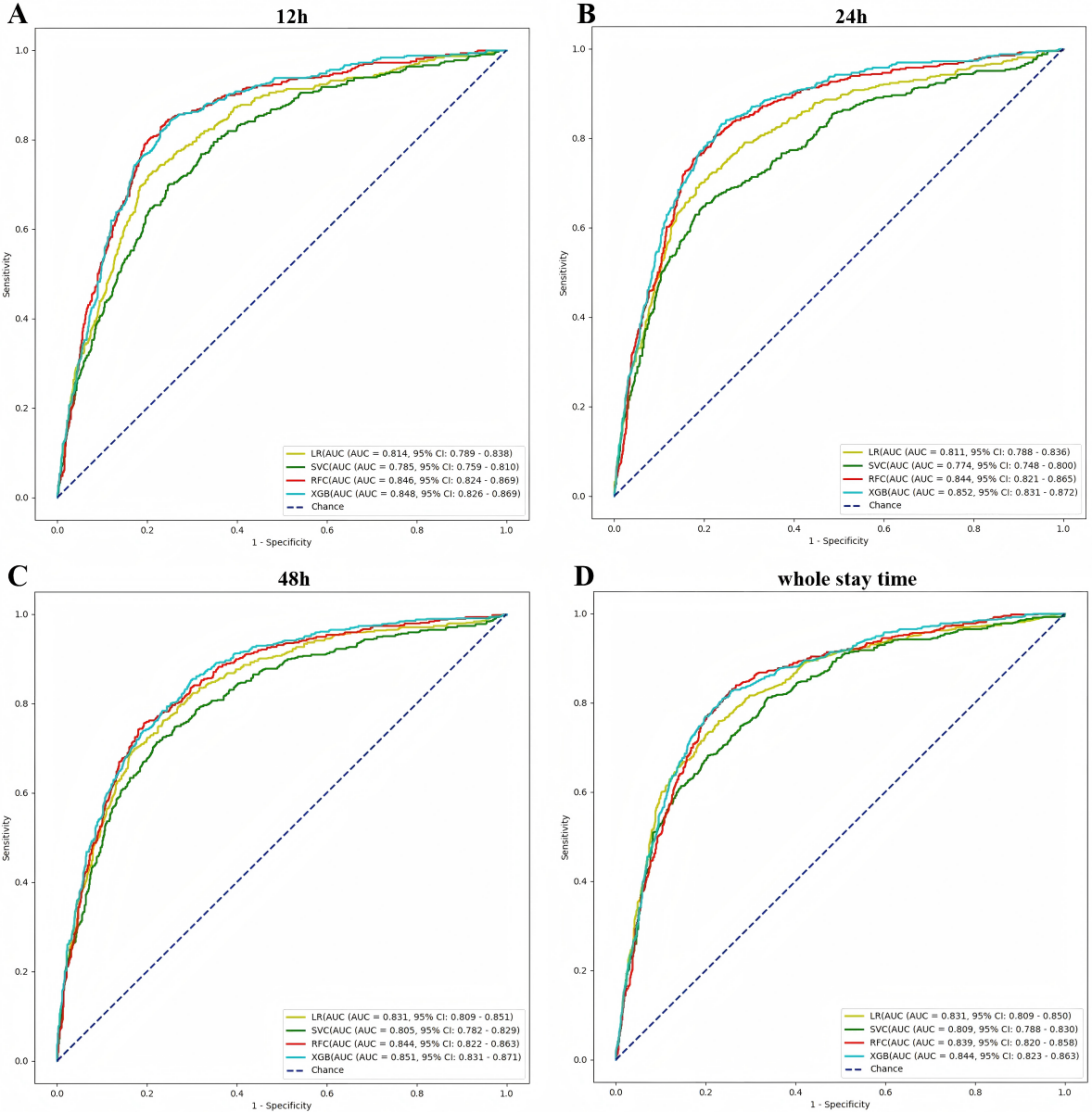
Receiver operating characteristic curves for all machine learning models in different prediction windows in the internal validation set. LR: logistic regression; SVC: support vector classifier; RFC: random forest classifier; XGB: extreme gradient boosting.

In total, 4 machine learning algorithms were used in the construction of prediction models for the occurrence of delirium in older adult ICU patients following surgery. Figure 2 illustrates the discriminative performance of the receiver operating characteristic curves of the 4 models across different prediction windows. The XGB model demonstrated the best prediction of postoperative delirium in older adult patients. The AUC values for the 4 prediction windows (12 h, 24 h, 48 h, and whole stay time) were 0.848 (95% CI 0.826-0.869), 0.852 (95% CI 0.831-0.872), 0.851 (95% CI 0.831-0.871), and 0.844 (95% CI 0.823-0.863), respectively. The RFC model also exhibits satisfactory prediction performance, although it is slightly inferior to that of the XGB model in general. The corresponding AUC values for the 4 prediction windows of the RFC model are 0.846 (95% CI 0.824-0.869), 0.844 (95% CI 0.821-0.865), 0.844 (95% CI 0.822-0.863), and 0.839 (95% CI 0.820-0.858), respectively. Overall, both models maintain a high performance in different prediction windows. The support vector classifier and LR models demonstrated significantly inferior performance compared to the first 2 models. Furthermore, the best-performing XGB models were validated using the following metrics: accuracy, sensitivity, specificity, positive predictive value, and negative predictive value, as illustrated in Table 2. The confusion matrices associated with these evaluation metrics are presented in Multimedia Appendix 4.

**Table 2:** The prediction performance of extreme gradient boosting models in different prediction windows in the internal validation set.

Prediction window	Accuracy, mean (95% CI)	Sensitivity, mean (95% CI)	Specificity, mean (95% CI)	PPV^a^, mean (95% CI)	NPV^b^, mean (95% CI)	AUC^c^, mean (95% CI)
12 h	0.811 (0.793-0.829)	0.674 (0.626-0.722)	0.845 (0.827-0.864)	0.524 (0.479-0.569)	0.911 (0.896-0.926)	0.848 (0.826-0.869)
24 h	0.809 (0.791-0.827)	0.703 (0.667-0.746)	0.842 (0.823-0.861)	0.580 (0.537-0.622)	0.902 (0.885-0.918)	0.852 (0.831-0.872)
48 h	0.795 (0.777-0.813)	0.708 (0.668-0.748)	0.827 (0.807-0.847)	0.603 (0.563-0.642)	0.884 (0.867-0.902)	0.851 (0.831-0.871)
Whole stay time	0.796 (0.778-0.814)	0.729 (0.692-0.766)	0.825 (0.804-0.846)	0.644 (0.607-0.682)	0.875 (0.856-0.894)	0.844 (0.823-0.863)

^a^PPV: positive predictive value.

^b^NPV: negative predictive value.

^c^AUC: area under the curve.

**Figure 3:**
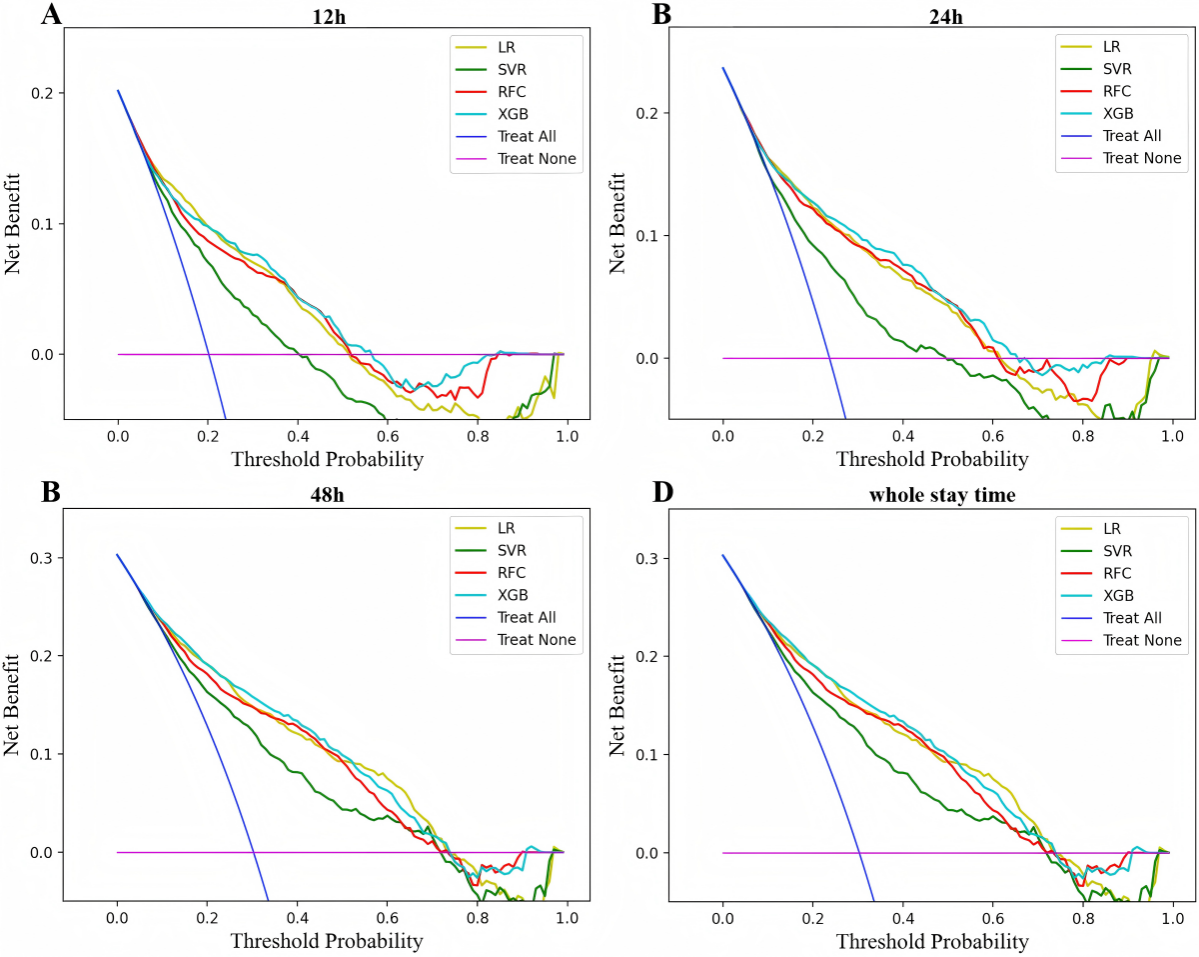
Decision curves for all machine learning models in different prediction windows in the internal validation set. LR: logistic regression; SVC: support vector classifier; RFC: random forest classifier; XGB: extreme gradient boosting.

We further evaluated the calibration performance of the model predictions by Brier score. As illustrated in Table 3, the XGB model demonstrates the best performance. The Brier scores for the XGB model in predicting delirium across different windows are 0.129, 0.136, 0.144, and 0.148, respectively, substantiating the reliability of our model. A decision curve is a tool used to evaluate the performance of a predictive model under different thresholds. As illustrated in Figure 3, for the internal validation dataset, the XGB model exhibits superior performance compared to other machine learning models across a range of thresholds for diverse prediction windows, with the RFC model exhibiting a marginal advantage in a few instances. When multiple evaluation metrics are considered, the XGB model emerges as the best algorithm.

**Table 3:** Brier scores for all machine learning models in different prediction windows in the internal validation set.

Prediction window	LR^a^	SVC^b^	RFC^c^	XGB^d^
12 h	0.135	0.159	0.134	0.129
24 h	0.144	0.171	0.142	0.136
48 h	0.146	0.169	0.148	0.144
Whole stay time	0.151	0.175	0.156	0.148

^a^LR: logistic regression.

^b^SVC: support vector classifier.

^c^RFC: random forest classifier.

^d^XGB: extreme gradient boosting.

**Figure 4:**
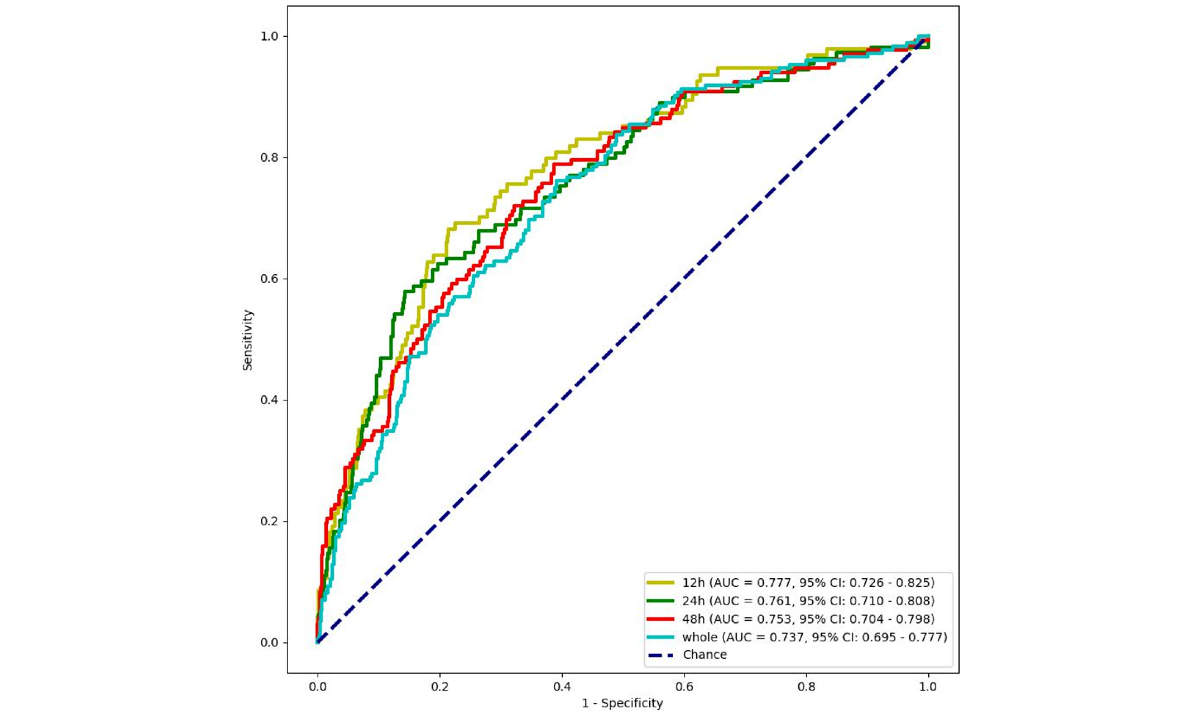
Receiver operating characteristic curves for extreme gradient boosting models in different prediction windows in the external validation set.

To evaluate the model's capacity for generalization and its ability to make predictions on new samples, an external validation of the XGB model was conducted using the eICU-CRD dataset from 208 different hospitals. With regard to the AUC values (Figure 4), the XGB model continues to demonstrate robust performance. The AUC values for the 4 prediction windows were 0.777 (95% CI 0.726-0.825), 0.761 (95% CI 0.710-0.808), 0.753 (95% CI 0.704-0.898), and 0.737 (95% CI 0.695-0.777), respectively. The comprehensive performance of the XGB model on the external validation set is presented in Table 4, and the associated confusion matrix plots are provided in Multimedia Appendix 5.

**Table 4:** The prediction performance of extreme gradient boosting models in different prediction windows in the external validation set.

Prediction window	Accuracy, mean (95% CI)	Sensitivity, mean (95% CI)	Specificity, mean (95% CI)	PPV^a^, mean (95% CI)	NPV^b^, mean (95% CI)	AUC^c^, mean (95% CI)
12 h	0.766 (0.735-0.797)	0.691 (0.598-0.785)	0.777 (0.744-0.810)	0.322 (0.257-0.386)	0.943 (0.923-0.963)	0.777 (0.726-0.825)
24 h	0.745 (0.841-0.894)	0.670 (0.581-0.758)	0.758 (0.724-0.793)	0.335 (0.272-0.398)	0.927 (0.904-0.950)	0.761 (0.710-0.808)
48 h	0.728 (0.695-0.761)	0.606 (0.523-0.689)	0.756 (0.721-0.791)	0.362 (0.299-0.425)	0.893 (0.866-0.921)	0.753 (0.704-0.898)
Whole stay time	0.705 (0.672-0.739)	0.599 (0.526-0.672)	0.739 (0.702-0.776)	0.424 (0.362-0.486)	0.852 (0.820-0.884)	0.737 (0.695-0.777)

^a^PPV: positive predictive value.

^b^NPV: negative predictive value.

^c^AUC: area under the curve.

### Variable Importance

**Figure 5:**
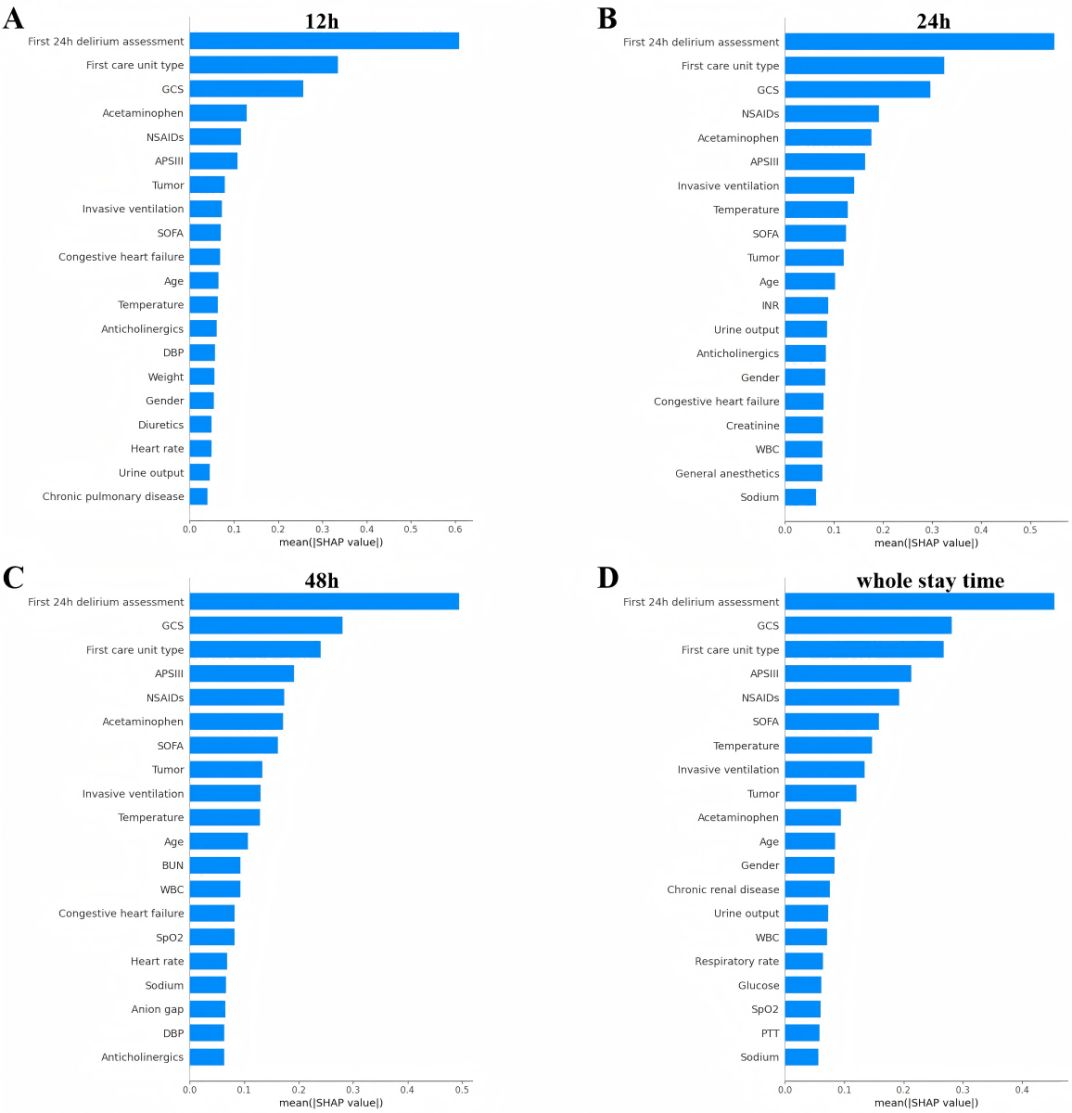
Feature importance ranking plot of the XGB machine learning models in different prediction windows (top 20 features). A, B, C, and D correspond to prediction windows of 12 h, 24 h, 48 h, and whole stay time, respectively. GCS: Glasgow Coma Scale; SOFA: Sequential Organ Failure Assessment; APSIII: Acute Physiology Score III; DBP: diastolic blood pressure; BUN: blood urea nitrogen; NSAIDS: nonsteroidal anti-inflammatory drugs; WBC: white blood cell; SpO2: oxygen saturation; XGB: extreme gradient boosting.

**Figure 6:**
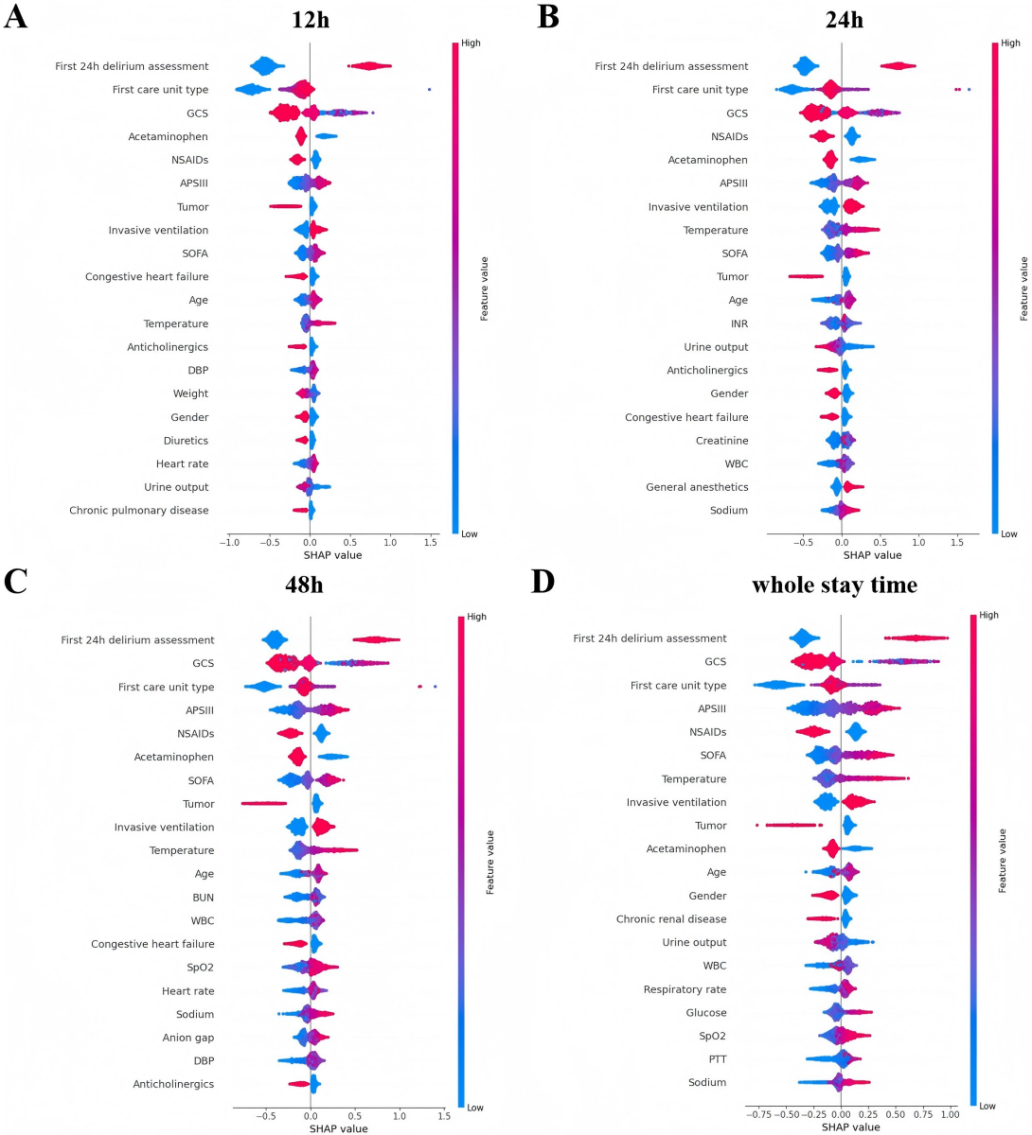
Shapley Additive Explanations (SHAP) summary plots of the XGB machine learning models in different prediction windows (top 20 features). A, B, C, and D correspond to prediction windows of 12 h, 24 h, 48 h, and whole stay time, respectively. Each point in the plot in a given case corresponds to the SHAP value of the element. The y-axis represents the feature, and the x-axis position indicates the SHAP value or the extent of the feature's impact on the prediction. The color of the points represents the actual values of the features, with purple indicating low values and red indicating high values. GCS: Glasgow Coma Scale; SOFA: Sequential Organ Failure Assessment; APSIII: Acute Physiology Score III; DBP: diastolic blood pressure; BUN: blood urea nitrogen; NSAIDs: nonsteroidal anti-inflammatory drugs; WBC: white blood cell; SpO2: oxygen saturation; XGB: extreme gradient boosting.

The results of the study demonstrated that each variable had a distinct predictive value with respect to the occurrence of delirium in older adult ICU patients who had undergone surgery. In order to identify the most influential features in the model, we plotted the feature importance rankings of the XGB model for different prediction windows, comprising the top 20 features (as illustrated in Figure 5). The ranking of features exhibits minor fluctuations across different prediction windows. In general, the most significant features were the first day's delirium assessment results, type of first care unit, minimum Glasgow Coma Scale (GCS) score, Acute Physiology Score III (APSIII), acetaminophen, and nonsteroidal anti-inflammatory drugs (NSAIDs). Furthermore, invasive ventilation, Sequential Organ Failure Assessment (SOFA) score, mean body temperature, age, tumor, and some laboratory metrics were also identified as relatively high-ranking features. The SHAP summary plot (Figure 6) complements the above ranking by illustrating the impact of each feature on the model output.

## Discussion

### Principal Results

This study presents the findings of a large-scale retrospective analysis conducted of an older adult ICU population with a high prevalence of postoperative delirium. The study developed the early prediction models for postoperative delirium in older adult ICU patients by training 4 major machine learning algorithms on the MIMIC-IV dataset. The XGB model, which exhibited the best performance in identifying delirium status during internal validation, demonstrated acceptable recognition and generalization capabilities in the external validation on the eICU-CRD dataset. Additionally, we identified that the most significant features were the first day's delirium assessment results, type of first care unit, minimum GCS score, APSIII, acetaminophen, and NSAIDs.

To the best of our knowledge, this is the inaugural predictive model to forecast postoperative delirium state in older adult patients in a windowed manner. The model leverages clinical data collected within the first 24 hours of ICU admission to predict delirium status at future intervals of 12, 24, and 48 h, as well as throughout the entire ICU stay. By retaining subjects with positive delirium assessment records during the observation window and incorporating delirium evaluation outcomes as model features, our framework enables nuanced analysis of delirium (eg, initial onset, recurrence, persistence, and remission) beyond binary classification. This tool assists clinicians in systematically evaluating patient status, optimizing clinical decision-making at critical junctures, and supplying preventive and personalized care to high-risk populations.

### Comparison With Prior Work

At present, the CAM-ICU (Confusion Assessment Method for the Intensive Care Unit) score is the most frequently used method for the clinical diagnosis of delirium, but it necessitates the administration of multiple assessments to ascertain a positive result [33]. Prior research has demonstrated that clinicians' forecasts of delirium progression are less precise than those of ICU delirium prediction models. This discrepancy may be attributed to various factors, including the lack of clinical experience among ICU personnel, the volume and intricacy of delirium assessment, and the dearth of attention devoted to delirium [34-37]. Although some predictive models for assessing the risk of postoperative delirium in older adult patients have been developed, existing studies still exhibit significant limitations in model construction and validation. First, most current models use training cohorts with limited sample sizes (n<2000) or lack rigorous external validation processes [20-23]. Some studies only use temporal split-validation within single medical institutions, rendering their conclusions of questionable generalizability [22]. Second, other models predominantly focus on specific surgical procedures (eg, cardiac surgery and hip fracture repair), failing to meet the multidisciplinary demands of clinical practice [38,39]. In contrast, this study developed a machine learning prediction model based on the MIMIC-IV database (n=6129) and conducted external validation using the multicenter eICU-CRD cohort encompassing 208 medical institutions across North America. The results demonstrate that our model achieved acceptable predictive performance for delirium status in both internal and external validations, with cross-institutional and cross-procedural stability validation providing robust evidence for clinical implementation.

The findings of this study reveal that the prevalence of postoperative delirium among older adult patients reached 40.6% in the MIMIC-IV database and 29.5% in the eICU-CRD, both of which are significantly higher than the rates reported in previous similar studies [40-42]. This discrepancy may stem from our inclusion of delirium-positive cases on the first day of admission, aiming to monitor the subsequent evolution of delirium status. Prior research has demonstrated that persistent or recurrent episodes of delirium are significantly associated with prolonged hospital stays and increased mortality rates [43]. Systematic clinical investigations and the establishment of effective monitoring mechanisms for this patient population hold substantial clinical value.

This study used SHAP to elucidate the intrinsic information of the XGB model, thereby offering a transparent rationale for personalized risk prediction of delirium. This facilitates a more intuitive comprehension of the influence of pivotal features and provides guidance for clinical decision-making. The present study identified 6 key factors most strongly associated with the onset of delirium in older adult patients in the ICU. These were the first day's delirium assessment results, type of first care unit, minimum GCS score, APSIII, acetaminophen, and NSAIDs. Furthermore, invasive ventilation, SOFA score, mean body temperature, age, and tumor were also identified as relatively high-ranking features. This means that certain highly predictive features identified in previous studies (such as age, invasive ventilation, temperature, SOFA score, GCS score, and type of first care unit) were corroborated in the present study [40,44,45]. APSIII, as part of the APACHE score, suggests a potential association with delirium [40]. In this study, the delirium status on the first day was consistently identified as the most critical predictive feature. Its prominent predictive value may be closely associated with the persistent characteristics of delirium pathophysiology. The onset of delirium on the first day often indicates that patients may have persistent underlying triggers that have not been effectively addressed (eg, metabolic imbalances, occult infections, or cerebral hemodynamic abnormalities). The effects of these pathological factors may drive persistent delirium episodes through cascade effects, thereby influencing subsequent clinical assessments. Incorporating this feature into predictive models can indirectly capture the continuity of latent pathological states. It is noteworthy that in the MIMIC-IV dataset of this study, 1812 individuals were identified as having delirium on day 1. But within the next 12 hours, 871 (48.1%) of these had moved to a nondelirious state. This finding suggests that while the first day’s delirium assessment holds significant predictive value, evaluation of delirium’s dynamic trajectory requires integration with multidimensional indicators.

This study demonstrates a significant association between the use of acetaminophen or NSAIDs and the risk of delirium occurrence. As antipyretic-analgesic agents widely used in clinical practice, their pharmacological mechanisms involve the inhibition of cyclooxygenase enzymes, thereby blocking prostaglandin biosynthesis and downregulating the hypothalamic thermoregulatory set point to achieve antipyretic effects [46,47]. Previous studies have revealed that prostaglandins not only participate in thermoregulation but also play a pivotal regulatory role in the pathophysiology of delirium through pathways affecting neurotransmitter release and neuroinflammatory responses [48]. These molecular findings corroborate clinical evidence from this study that identifies abnormal body temperature as an independent risk factor for delirium. This suggests that when developing body temperature management strategies for delirium patients, the neuroregulatory effects of such medications should be taken into consideration. At the same time, this study incorporated various chronic comorbidities to investigate the risk factors for delirium. The results revealed that the most significant association with delirium was observed in tumor patients. This correlation may stem from tumor-related metabolic disturbances leading to neurotransmitter imbalances, neurotoxic effects of anti-tumor treatments (such as chemotherapy drugs and radiotherapy), as well as the prevalent pain-induced stress and sleep disturbances among advanced-stage tumor patients [49,50]. Other predictors such as blood sodium, oxygen saturation, blood urea nitrogen, blood pressure, urine output, and platelets have been validated by similar studies or predictive models [51-53].

### Strengths and Limitations

It is important to note that our study has several notable contributions and strengths. First, in order to ensure the quality and quantity of the data, 2 widely recognized high-quality databases were used: MIMIC-IV database and eICU-CRD. These databases are characterized by a large sample size and rich clinical data. This approach guarantees the internal validity of model training. More importantly, through rigorous external validation procedures, it confirms the model's generalizability across diverse hospital populations, significantly enhancing the clinical applicability of the research findings. Second, distinct from traditional single time-window prediction models, this study developed 4 progressive predictive models with different prediction windows (12 h, 24 h, 48 h, and whole stay time). This innovative design enables both short-term and long-term prediction of delirium status, which is crucial for improving care continuity and optimizing resource allocation in resource-constrained clinical environments. Last but not least, all predictive variables were derived from routine monitoring data collected within the first 24 hours of ICU admission, requiring no additional testing for real-time implementation. Notably, this study incorporates delirium assessment features within the first 24 hours of ICU admission for the first time, considering the persistent clinical monitoring value of delirium recurrence, continuation, and improvement in real-world scenarios. The early and accurate prediction of delirium status achieved in this research empowers clinicians to adjust treatment strategies with enhanced temporal efficiency.

It is important to acknowledge that our study has certain limitations. First, our study was implemented and validated retrospectively, and therefore further prospective intervention studies are required to validate the performance of the model. Second, during the external validation process, the research team was unable to fully reproduce the data screening criteria of the MIMIC-IV dataset within the eICU-CRD due to inherent structural differences between the databases. The particularly critical issue is that the eICU-CRD lacks explicit documentation of surgical time stamps, which hinders our ability to establish precise temporal correlations between ICU data and surgical events as effectively as we do in the MIMIC-IV database. This limitation ultimately leads to performance degradation of the model during external validation. Third, there is a possibility of selection bias and interpretive bias, as only variables that were available in all cohorts and easily extracted from the database were selected in order to ensure the accuracy and validity of the data. Furthermore, patients who did not have sufficiently valid delirium assessment data (65% of total ICU admissions after the initial screening in the MIMIC-IV dataset) were excluded. Fourth, the European and United States databases were sourced for this study due to the inherent limitations of genetically distinct populations with unique attributes that prevent the predictive models derived from these databases from being generalized to other populations. Fifth, state-of-the-art approaches to model interpretation, including SHAP and its alternatives, fail to account for dependencies of the nonlinearities between features, which inevitably introduces correlation bias [54,55].

### Conclusions

In this study, we constructed and validated a high-performance prediction model for postoperative delirium in older adult ICU patients. This model can predict delirium state in the subsequent 12 h, 24 h, 48 h, and whole stay time using clinical data obtained within 24 hours of ICU admission. It enables clinicians to promptly identify older adult patients at elevated risk of delirium, thus facilitating the implementation of targeted and individualized interventions to enhance prognosis and optimize management strategies while rationalizing health care resources.

## Data Availability

The datasets used and analyzed during the current study are available from the corresponding author on reasonable request.

## Appendix

Multimedia Appendix 1 Baseline characteristics of patients with and those without delirium in the 12-h prediction window.

Multimedia Appendix 2 Baseline characteristics of patients with and those without delirium in the 24-h prediction window.

Multimedia Appendix 3 Baseline characteristics of patients with and those without delirium in the 48-h prediction window.

Multimedia Appendix 4 Confusion matrix of extreme gradient boosting models for different prediction windows in the internal validation set.

Multimedia Appendix 5 Confusion matrix of extreme gradient boosting models for different prediction windows in the external validation set.

## Abbreviations

APSIII: Acute Physiology Score III
AUC: area under the curve
CAM-ICU: Confusion Assessment Method for the Intensive Care Unit
eICU-CRD: eICU Collaborative Research Database
GCS: Glasgow Coma Scale
ICU: intensive care unit
LR: logistic regression
MIMIC-IV: Medical Information Marketplace for Intensive Care IV
NSAID: nonsteroidal antiinflammatory drug
RFC: random forest classifier
SHAP: Shapley Additive Explanations
SOFA: sequential organ failure assessment
XGB: extreme gradient boosting
