# Supplementary material for: Development of a Machine Learning–Based Predictive Model for Postoperative Delirium in Older Adult Intensive Care Unit Patients: Retrospective Study
Source: J Med Internet Res. 2025 Jun 19;27:e67258. doi: 10.2196/67258 (PMC12226778; doi:10.2196/67258)
Supplement: Multimedia Appendix 1 [file jmir_v27i1e67258_app1.docx]

Multimedia Appendix 1: Baseline characteristics of patients with and those without delirium in the 12-h prediction window.

| Patients Characteristics | MIMIC-IV^a^ cohort | | | eICU-CRD^b^ cohort | | |
| --- | --- | --- | --- | --- | --- | --- |
|  | No Delirium  (n=4894) | Delirium  (n=1235) | P Value | No Delirium  (n=615) | Delirium  (n=94) | P Value |
| **Demographic data** |  |  |  |  |  |  |
| Age (years), median (IQR) | 75.0 (70.0-82.0) | 78.0 (71.0-84.0) | <.001 | 74.0 (69.0-80.0) | 76.0 (70.0-83.0) | .20 |
| Male gender, n (%) | 2739.0 (56.0) | 625.0 (50.6) | <.001 | 312.0 (50.7) | 52.0 (55.3) | .41 |
| Weight (kg), median (IQR) | 78.4 (66.3-91.5) | 75.0 (63.1-88.9) | <.001 | 77.6 (65.8-91.2) | 73.1 (60.1-90.2) | .11 |
| Race, n (%) |  |  | <.001 |  |  | .43 |
| Black | 348.0 (7.1) | 127.0 (10.3) |  | 74.0 (12.0) | 12.0 (12.8) |  |
| White | 3471.0 (70.9) | 776.0 (62.8) |  | 488.0 (79.3) | 71.0 (75.5) |  |
| Asian | 118.0 (2.4) | 17.0 (1.4) |  | 2.0 (0.3) | 1.0 (1.1) |  |
| Hispanic | 79.0 (1.6) | 33.0 (2.7) |  | 24.0 (3.9) | 3.0 (3.2) |  |
| Other or unknown | 878.0 (17.9) | 282.0 (22.8) |  | 27.0 (4.4) | 7.0 (7.4) |  |
| **First care unit type, n (%)** |  |  | <.001 |  |  | .09 |
| Cardiovascular ICU^c^ | 2078.0 (42.5) | 234.0 (18.9) |  | 128.0 (20.8) | 22.0 (23.4) |  |
| Neurological ICU | 507.0 (10.4) | 199.0 (16.1) |  | 95.0 (15.4) | 22.0 (23.4) |  |
| Other ICU | 2309.0 (47.2) | 802.0 (64.9) |  | 392.0 (63.7) | 50.0 (53.2) |  |
| **First 24h delirium assessment, n (%)** |  |  | <.001 |  |  | <.001 |
| Negative | 4023.0 (82.2) | 294.0 (23.8) |  | 569.0 (92.5) | 31.0 (33.0) |  |
| Positive | 871.0 (17.8) | 941.0 (76.2) |  | 46.0 (7.5) | 63.0 (67.0) |  |
| **Vital signs, median (IQR)** |  |  |  |  |  |  |
| Heart rate, beats/min | 79.7 (71.3-89.6) | 82.4 (73.4-94.6) | <.001 | 83.2 (74.5-92.4) | 85.9 (78.8-97.8) | .006 |
| Systolic blood pressure, mmHg | 115.4 (106.6-126.6) | 115.9 (106.9-127.7) | .05 | 118.5 (107.8-131.2) | 119.2 (106.5-129.1) | .67 |
| Diastolic blood pressure,mmHg | 58.7 (53.0-65.5) | 60.2 (54.2-66.6) | <.001 | 60.6 (55.5-67.0) | 60.8 (55.3-67.2) | .64 |
| Mean blood pressure, mmHg | 58.7 (53.0-65.5) | 60.2 (54.2-66.6) | <.001 | 77.9 (70.4-85.1) | 76.9 (70.3-84.9) | .82 |
| Respiratory rate, beats/min | 18.3 (16.5,20.5) | 18.9 (16.9,21.4) | <.001 | 17.6 (15.7-20.0) | 17.8 (15.7-21.4) | .60 |
| Temperature, $℃$ | 36.8 (36.6-37.0) | 36.9 (36.7-37.2) | <.001 | 36.8 (36.6-37.1) | 36.9 (36.6-37.2) | 0.99 |
| Oxygen saturation, % | 97.1 (95.8-98.3) | 97.6 (96.2-98.8) | <.001 | 97.3 (95.8-98.4) | 97.3 (96.1-98.6) | .48 |
| **Laboratory results, median (IQR)** |  |  |  |  |  |  |
| Hematocrit, % | 31.6 (27.9-35.6) | 31.9 (27.5-36.0) | .64 | 31.1 (27.3-34.4) | 30.9 (27.9-34.4) | .52 |
| Hemoglobin, g/dL | 10.3 (9.1-11.7) | 10.3 (8.8-11.7) | .08 | 10.3 (9.0-11.4) | 10.2 (8.9-11.3) | .99 |
| Platelet, 10^9^/L | 175.6 (132.5-230.0) | 179.0 (131.3-240.5) | .32 | 187.0 (140.3-235.0) | 175.5 (121.5-253.5) | .69 |
| White blood cell, 10^9^/L | 11.2 (8.4-14.9) | 12.0 (9.1-15.7) | <.001 | 11.7 (9.2-15.3) | 12.2 (9.4-16.5) | .39 |
| Anion gap, mmol/L | 13.3 (11.5-15.5) | 14.3 (12.3-17.0) | <.001 | 10.4( 8.0-13.0) | 11.5 (8.3-14.6) | .04 |
| Blood urea nitrogen, mg/dL | 19.7 (14.5-30.0) | 24.7 (16.7-40.5) | <.001 | 19.0 (13.0-28.5) | 22.3 (14.0-34.0) | .06 |
| Calcium, mg/dL | 8.3 (8.0-8.7) | 8.4 (7.9-8.8) | .34 | 8.2 (7.8-8.5) | 8.1 (7.8-8.6) | .95 |
| Chloride, mmol/L | 105.0 (101.3-107.7) | 104.7 (101.0-108.0) | .62 | 105.0 (102.0-108.0) | 106.3 (102.9-110.1) | .02 |
| Creatinine, mg/dL | 1.0 (0.8-1.4) | 1.1 (0.8-1.8) | <.001 | 1.0 (0.8-1.4) | 1.1 (0.8-1.6) | .06 |
| Glucose, mg/dL | 128.0 (109.0-152.3) | 135.5 (111.0-173.5) | <.001 | 139.2 (117.8-161.3) | 137.5 (116.5-162.0) | .67 |
| Sodium, mmol/L | 138.4 (136.0-140.5) | 139.2 (136.0-142.0) | <.001 | 138.5 (136.0-141.0) | 140.0 (137.0-143.5) | .002 |
| Potassium, mmol/L | 4.2 (3.9-4.5) | 4.2 (3.8-4.5) | .41 | 4.2 (3.8-4.6) | 4.1 (3.8-4.4) | .35 |
| International normalized ratio | 1.3 (1.2-1.4) | 1.3 (1.2-1.5) | .004 | 1.4 (1.2-1.6) | 1.5 (1.3-1.9) | <.001 |
| Prothrombin time, s | 14.1 (12.5-15.4) | 14.4 (12.4-16.3) | .003 | 16.0 (14.1-18.3) | 17.9 (15.0-21.2) | <.001 |
| partial thromboplastin time, s | 31.4 (27.9-38.3) | 31.3 (27.3-38.6) | .21 | 35.5 (35.4-35.6) | 35.4 (35.3-35.6) | .08 |
| Urine output, ml | 1450.0 (960.0-2125.0) | 1224.0 (718.2-1845.0) | <.001 | 1330.0 (870.0-1775.0) | 1003.5 (555.0-1830.0) | .02 |
| **Comorbidity, n (%)** |  |  |  |  |  |  |
| Hypertension | 3840.0 (78.5) | 997.0 (80.7) | .08 | 114.0 (18.5) | 16.0 (17.0) | .72 |
| Diabetes | 1606.0 (32.8) | 470.0 (38.1) | <.001 | 86.0 (14.0) | 10.0 (10.6) | .38 |
| Congestive heart failure | 1691.0 (34.6) | 493.0 (39.9) | <.001 | 51.0 (8.3) | 5.0 (5.3) | .32 |
| Chronic renal disease | 1221.0 (24.9) | 397.0 (32.1) | <.001 | 53.0 (8.6) | 5.0 (5.3) | .28 |
| Chronic liver disease | 401.0 (8.2) | 111.0 (9.0) | .37 | 6.0 (1.0) | 4.0 (4.3) | .03 |
| Chronic pulmonary disease | 1309.0 (26.7) | 359.0 (29.1) | .10 | 56.0 (9.1) | 11.0 (11.7) | .42 |
| Peptic ulcer disease | 153.0 (3.1) | 43.0 (3.5) | .53 | 4.0 (0.7) | 1.0 (1.1) | .51 |
| Tumor | 807.0 (16.5) | 179.0 (14.5) | .09 | 133.0 (21.6) | 11.0 (11.7) | .03 |
| Dementia | 153.0 (3.1) | 189.0 (15.3) | <.001 | 7.0 (1.1) | 4.0 (4.3) | .05 |
| **Score, median (IQR)** |  |  |  |  |  |  |
| GCS^d^ | 15.0 (14.0-15.0) | 14.0 (12.0-15.0) | <.001 | 14.0 (11.0-15.0) | 12.0 (7.0-14.0) | <.001 |
| SOFA^e^ | 4.0 (2.0-6.0) | 6.0 (4.0-9.0) | <.001 | 5.0 (4.0-7.0) | 7.0 (5.0-9.0) | <.001 |
| APSIII^f^ | 39.0 (30.0-50.0) | 49.0 (38.0-64.0) | <.001 | 40.0 (30.0-54.0) | 50.5 (38.0-67.0) | <.001 |
| **Treatment measures, n (%)** |  |  |  |  |  |  |
| Renal replacement therapy | 161.0 (3.3) | 69.0 (5.6) | <.001 | 20.0 (3.3) | 4.0 (4.3) | .55 |
| Invasive ventilation | 2069.0 (42.3) | 816.0 (66.1) | <.001 | 233.0 (37.9) | 48.0 (51.1) | .02 |
| Acetaminophen | 3768.0 (77.0) | 794.0 (64.3) | <.001 | 312.0 (50.7) | 58.0 (61.7) | .05 |
| Anticholinergics | 1548.0 (31.6) | 366.0 (29.6) | .18 | 43.0 (7.0) | 12.0 (12.8) | .05 |
| Anticoagulants | 3200.0 (65.4) | 886.0 (71.7) | <.001 | 226.0 (36.7) | 30.0 (31.9) | .36 |
| Antihistamines | 255.0 (5.2) | 49.0 (4.0) | .07 | 38.0 (6.2) | 4.0 (4.3) | .46 |
| Antipsychotics | 195.0 (4.0) | 166.0 (13.4) | <.001 | 7.0 (1.1) | 4.0 (4.3) | .05 |
| Benzodiazepines | 752.0 (15.4) | 188.0 (15.2) | .90 | 101.0 (16.4) | 22.0 (23.4) | .10 |
| Diuretics | 1994.0 (40.7) | 458.0 (37.1) | .02 | 149.0 (24.2) | 23.0 (24.5) | .96 |
| General anesthetics | 1886.0 (38.5) | 711.0 (57.6) | <.001 | 79.0 (12.8) | 21.0 (22.3) | .01 |
| NSAIDs^g^ | 2503.0 (51.1) | 448.0 (36.3) | <.001 | 121.0 (19.7) | 27.0 (28.7) | .04 |
| Opioids | 4224.0 (86.3) | 1093.0 (88.5) | .04 | 318.0 (51.7) | 57.0 (60.6) | .11 |
| Vasopressors | 2432.0 (49.7) | 659.0 (53.4) | .02 | 107.0 (17.4) | 28.0 (29.8) | .004 |

^a^MIMIC-IV: Medical Information Marketplace for Intensive Care IV.

^b^eICU-CRD: eICU Collaborative Research Database.

^c^ICU: intensive care unit.

^d^GCS: Glasgow Coma Scale.

^e^SOFA: Sequential Organ Failure Assessment.

^f^APSIII: Acute Physiology Score III.

^g^NSAIDs: Nonsteroidal Antiinflammatory Drugs.
