# Supplementary material for: Development of a Machine Learning–Based Predictive Model for Postoperative Delirium in Older Adult Intensive Care Unit Patients: Retrospective Study
Source: J Med Internet Res. 2025 Jun 19;27:e67258. doi: 10.2196/67258 (PMC12226778; doi:10.2196/67258)
Supplement: Multimedia Appendix 2 [file jmir_v27i1e67258_app2.docx]

Multimedia Appendix 2: Baseline characteristics of patients with and those without delirium in the 24-h prediction window.

| Patients Characteristics | MIMIC-IV^a^ cohort | | | eICU-CRD^b^ cohort | | |
| --- | --- | --- | --- | --- | --- | --- |
|  | No Delirium  (n=4679) | Delirium  (n=1450) | P Value | No Delirium  (n=600) | Delirium  (n=109) | P Value |
| **Demographic data** |  |  |  |  |  |  |
| Age (years), median (IQR) | 75.0 (70.0-82.0) | 77.0 (71.0-84.0) | <.001 | 74.0 (69.0-80.0) | 76.0 (70.0-83.0) | .14 |
| Male gender, n (%) | 2618.0 (56.0) | 746.0 (51.4) | .003 | 304.0 (50.7) | 60.0 (55.0) | .40 |
| Weight (kg), median (IQR) | 78.5 (66.5-91.7) | 75.0 (63.0-88.7) | <.001 | 78.0 (65.8-91.2) | 72.5 (62.0-90.2) | .09 |
| Race, n (%) |  |  | <.001 |  |  | .36 |
| Black | 331.0 (7.1) | 144.0 (9.9) |  | 69.0 (11.5) | 17.0 (15.6) |  |
| White | 3331.0 (71.2) | 916.0 (63.2) |  | 479.0 (79.8) | 80.0 (73.4) |  |
| Asian | 115.0 (2.5) | 20.0 (1.4) |  | 2.0 (0.3) | 1.0 (0.9) |  |
| Hispanic | 78.0 (1.7) | 34.0 (2.3) |  | 23.0 (3.8) | 4.0 (3.7) |  |
| Other or unknown | 824.0 (17.6) | 336.0 (23.2) |  | 27.0 (4.5) | 7.0 (6.4) |  |
| **First care unit type, n (%)** |  |  | <.001 |  |  | .16 |
| Cardiovascular ICU^c^ | 2024.0 (43.3) | 288.0 (19.9) |  | 125.0 (20.8) | 25.0 (22.9) |  |
| Neurological ICU | 468.0 (10.0) | 238. 0 (16.4) |  | 93.0 (15.5) | 24.0 (22.0) |  |
| Other ICU | 2187.0 (46.7) | 924.0 (63.7) |  | 382.0 (63.7) | 60.0 (55.0) |  |
| **First 24h delirium assessment, n (%)** |  |  | <.001 |  |  | <.001 |
| Negative | 3915.0 (83.7) | 402.0 (27.7) |  | 556.0 (92.7) | 44.0 (40.4) |  |
| Positive | 764.0 (16.3) | 1048.0 (72.3) |  | 44.0 (7.3) | 65.0 (59.6) |  |
| **Vital signs, median (IQR)** |  |  |  |  |  |  |
| Heart rate, beats/min | 79.7 (71.3-89.6) | 82.2 (73.0-94.0) | <.001 | 83.1 (74.5-92.1) | 85.8 (78.5-97.6) | .006 |
| Systolic blood pressure, mmHg | 115.4 (106.6-126.5) | 116.1 (107.0-128.2) | .02 | 118.5 (108.0-131.2) | 119.3 (105.3-129.3) | .58 |
| Diastolic blood pressure, mmHg | 58.7 (53.0-65.5) | 60.1 (53.9-66.5) | <.001 | 60.6 (55.5-67.1) | 61.0 (55.1-67.0) | .79 |
| Mean blood pressure, mmHg | 75.1 (69.9-82.1) | 76.1 (70.7-83.2) | <.001 | 78.0 (70.4-85.0) | 77.4 (70.2-85.9) | .82 |
| Respiratory rate, beats/min | 18.2 (16.5-20.5) | 18.9 (16.9-21.4) | <.001 | 17.6 (15.7-20.0) | 17.7 (15.6-21.5) | .71 |
| Temperature, $℃$ | 36.8 (36.6-37.0) | 36.9 (36.7-37.2) | <.001 | 36.8 (36.6-37.1) | 36.9 (36.6-37.2) | .93 |
| Oxygen saturation, % | 97.1 (95.7-98.3) | 97.6 (96.2-98.8) | <.001 | 97.3 (95.8-98.4) | 97.7 (96.2-98.7) | .11 |
| **Laboratory results, median (IQR)** |  |  |  |  |  |  |
| Hematocrit, % | 31.5 (28.0-35.5) | 32.0 (27.5-36.0) | .51 | 31.2 (27.6-34.4) | 30.5 (27.1-34.4) | .88 |
| Hemoglobin, g/dL | 10.3 (9.1-11.7) | 10.4 (8.9-11.7) | .16 | 10.3 (9.1-11.4) | 10.1 (8.8-11.4) | .55 |
| Platelet, 10^9^/L | 176.0 (132.5- 30.0) | 176.2 (131.3-240.0) | .47 | 187.0 (140.2-235.0) | 175.0 (134.0-248.0) | .62 |
| White blood cell, 10^9^/L | 11.1 (8.4-14.9) | 11.9 (9.1-15.7) | <.001 | 11.8 (9.3-15.3) | 12.0 (9.3-16.3) | .66 |
| Anion gap, mmol/L | 13.3 (11.5-15.5) | 14.3 (12.3-17.0) | <.001 | 10.5 (8.0-13.0) | 11.3 (8.5-14.5) | .08 |
| Blood urea nitrogen, mg/dL | 19.5 (14.3-30.0) | 24.0 (16.5-39.5) | <.001 | 19.0 (13.0-28.0) | 22.0 (14.5-34.0) | .02 |
| Calcium, mg/dL | 8.3 (8.0-8.7) | 8.3 (7.9-8.7) | .47 | 8.2 (7.8-8.5) | 8.1 (7.8-8.6) | .72 |
| Chloride, mmol/L | 104.7 (101.0-107.5) | 105.0 (101.0-108.3) | .05 | 105.0 (102.0-108.0) | 106.0 (102.8-110.0) | .02 |
| Creatinine, mg/dL | 1.0 (0.8-1.4) | 1.1 (0.8-1.7) | <.001 | 1.0 (0.8-1.4) | 1.1 (0.8-1.6) | .01 |
| Glucose, mg/dL | 128.0 (109.0-152.0) | 134.8 (111.0-172.0) | <.001 | 139.3 (118.0-161.2) | 137.0 (116.5-162.0) | .68 |
| Sodium, mmol/L | 138.3 (136.0-140.5) | 139.3 (136.3-142.0) | <.001 | 138.5 (136.0-141.0) | 140.0 (137.0-143.0) | .003 |
| Potassium, mmol/L | 4.2 (3.9-4.5) | 4.2 (3.8-4.5) | .28 | 4.2 (3.8-4.5) | 4.2 (3.9-4.5) | .67 |
| International normalized ratio | 1. 3 (1.2-1.4) | 1.3 (1.2-1.5) | .03 | 1.4 (1.2-1.6) | 1.5 (1.2-1.9) | .03 |
| Prothrombin time, s | 14.1 (12.6-15.4) | 14.3 (12.4-16.1) | .03 | 16.1 (14.2-18.4) | 17.0 (14.0-20.7) | .02 |
| partial thromboplastin time, s | 31.4 (27.8-38.3) | 31.4 (27.5-38.5) | .24 | 35.5 (35.4-35.6) | 35.4 (35.3-35.6) | .02 |
| Urine output, ml | 1465.0 (975.0-2145.0) | 1219.0 (730.0-1820.0) | <.001 | 1332.5 (874.8-1772.8) | 1020.0 (625.0-1895.0) | .02 |
| **Comorbidity, n (%)** |  |  |  |  |  |  |
| Hypertension | 3666.0 (78.4) | 1171.0 (80.8) | .05 | 111.0 (18.5) | 19.0 (17.4) | .79 |
| Diabetes | 1532.0 (32.7) | 544.0 (37.5) | <.001 | 81.0 (13.5) | 15.0 (13.8) | .94 |
| Congestive heart failure | 1617.0 (34.6) | 567.0 (39.1) | .002 | 51.0 (8.5) | 5.0 (4.6) | .16 |
| Chronic renal disease | 1159.0 (24.8) | 459.0 (31.7) | <.001 | 50.0 (8.3) | 8.0 (7.3) | .73 |
| Chronic liver disease | 384.0 (8.2) | 128.0 (8.8) | .46 | 6.0 (1.0) | 4.0 (3.7) | .05 |
| Chronic pulmonary disease | 1239.0 (26.5) | 429.0 (29.6) | .02 | 55.0 (9.2) | 12.0 (11.0) | .51 |
| Peptic ulcer disease | 1532.0 (32.7) | 544.0 (37.5) | <.001 | 4.0 (0.7) | 1.0(0.9) | .57 |
| Tumor | 778.0 (16.6) | 208.0 (14.3) | .04 | 128.0 (21.3) | 16.0 (14.7) | .11 |
| Dementia | 133.0 (2.8) | 209.0 (14.4) | <.001 | 7.0 (1.2) | 4.0 (3.7) | .07 |
| **Score, median (IQR)** |  |  |  |  |  |  |
| GCS^d^ | 15.0 (14.0-15.0) | 14.0 (12.0-15.0) | <.001 | 14.0 (11.0-15.0) | 12.0 (8.0-14.0) | <.001 |
| SOFA^e^ | 4.0 (2.0-6.0) | 6.0 (3.0-9.0) | <.001 | 5.0 (4.0-7.0) | 7.0 (5.0-9.0) | <.001 |
| APSIII^f^ | 39.0 (30.0-50.0) | 49.0 (38.0-63.0) | <.001 | 40.0 (30.0-53.0) | 51.0 (39.0-70.0) | <.001 |
| **Treatment measures, n (%)** |  |  |  |  |  |  |
| Renal replacement therapy | 151.0 (3.2) | 79.0 (5.4) | <.001 | 20.0 (3.3) | 4.0 (3.7) | .78 |
| Invasive ventilation | 1928.0 (41.2) | 957.0 (66.0) | <.001 | 226.0 (37.7) | 55.0 (50.5) | .01 |
| Acetaminophen | 3616.0 (77.3) | 946.0 (65.2) | <.001 | 303.0 (50.5) | 67.0 (61.5) | .04 |
| Anticholinergics | 1481.0 (31.7) | 433.0 (29.9) | .20 | 40.0 (6.7) | 15.0 (13.8) | .01 |
| Anticoagulants | 3057.0 (65.3) | 1029.0 (71.0) | <.001 | 217.0 (36.2) | 39.0 (35.8) | .94 |
| Antihistamines | 248.0 (5.3) | 56.0 (3.9) | 0.28 | 37.0 (6.2) | 5.0 (4.6) | .52 |
| Antipsychotics | 183.0 (3.9) | 178.0 (12.3) | <.001 | 7.0 (1.2) | 4.0 (3.7) | .07 |
| Benzodiazepines | 720.0 (15.4) | 220.0 (15.2) | .84 | 99.0 (16.5) | 24.0 (22.0) | .16 |
| Diuretics | 1907.0 (40.8) | 545.0 (37.6) | .03 | 145.0 (24.2) | 27.0 (24.8) | .89 |
| General anesthetics | 1762.0 (37.7) | 835.0 (57.6) | <.001 | 77.0 (12.8) | 23.0 (21.1) | .02 |
| NSAIDs^g^ | 2422.0 (51.8) | 529.0 (36.5) | <.001 | 120.0 (20.0) | 28.0 (25.7) | .18 |
| Opioids | 4028.0 (86.1) | 1289.0 (88.9) | .006 | 306.0 (51.0) | 69.0 (63.3) | .02 |
| Vasopressors | 2320.0 (49.6) | 771.0 (53.2) | .02 | 103.0 (17.2) | 32.0 (29.4) | .003 |

^a^MIMIC-IV: Medical Information Marketplace for Intensive Care IV.

^b^eICU-CRD: eICU Collaborative Research Database.

^c^ICU: intensive care unit.

^d^GCS: Glasgow Coma Scale.

^e^SOFA: Sequential Organ Failure Assessment.

^f^APSIII: Acute Physiology Score III.

^g^NSAIDs: Nonsteroidal Antiinflammatory Drugs.
