# Supplementary material for: Development of a Machine Learning–Based Predictive Model for Postoperative Delirium in Older Adult Intensive Care Unit Patients: Retrospective Study
Source: J Med Internet Res. 2025 Jun 19;27:e67258. doi: 10.2196/67258 (PMC12226778; doi:10.2196/67258)
Supplement: Multimedia Appendix 3 [file jmir_v27i1e67258_app3.docx]

Multimedia Appendix 3: Baseline characteristics of patients with and those without delirium in the 48-h prediction window.

| Patients Characteristics | MIMIC-IV^a^ cohort | | | eICU-CRD^b^ cohort | | |
| --- | --- | --- | --- | --- | --- | --- |
|  | No Delirium  (n=4471) | Delirium  (n=1658) | P Value | No Delirium  (n=577) | Delirium  (n=132) | P Value |
| **Demographic data** |  |  |  |  |  |  |
| Age (years), median (IQR) | 75.0 (70.0-82.0) | 77.0 (71.0-84.0) | <.001 | 74.0 (69.0-80.0) | 76.0 (70.5-82.5) | .08 |
| Male gender, n (%) | 2509.0 (56.1) | 855.0 (51.6) | <.001 | 289.0 (50.1) | 75.0 (56.8) | .16 |
| Weight (kg), median (IQR) | 78.5 (66.6-91.6) | 75.4 (63.0-89.1) | <.001 | 77.6 (65.8-91.2) | 75.0 (62.1-90.8) | .35 |
| Race, n (%) |  |  | <.001 |  |  | .46 |
| Black | 316.0 (7.1) | 159.0 (9.6) |  | 66.0 (11.4) | 20.0 (15.2) |  |
| White | 3200.0 (71.6) | 1047.0 (63.1) |  | 460.0 (79.7) | 99.0 (75.0) |  |
| Asian | 112.0 (2.5) | 23.0 (1.4) |  | 2.0 (0.3) | 1.0 (0.8) |  |
| Hispanic | 77.0 (1.7) | 35.0 (2.1) |  | 23.0 (4.0) | 4.0 (3.0) |  |
| Other or unknown | 766.0 (17.1) | 394.0 (23.8) |  | 26.0 (4.5) | 8.0 (6.1) |  |
| **First care unit type, n (%)** |  |  | <.001 |  |  | .06 |
| Cardiovascular ICU^c^ | 1972.0 (44.1) | 340.0 (20.5) |  | 118.0 (20.5) | 32.0 (24.2) |  |
| Neurological ICU | 445.0 (10.0) | 261.0 (15.7) |  | 88.0 (15.3) | 29.0 (22.0) |  |
| Other ICU | 2054.0 (45.9) | 1057.0 (63.8) |  | 371.0 (64.3) | 71.0 (53.8) |  |
| **First 24h delirium assessment, n (%)** |  |  | <.001 |  |  | <.001 |
| Negative | 3788.0 (84.7) | 529.0 (31.9) |  | 537.0 (93.1) | 63.0 (47.7) |  |
| Positive | 683.0 (15.3) | 1129.0 (68.1) |  | 40.0 (6.9) | 69.0 (52.3) |  |
| **Vital signs, median (IQR)** |  |  |  |  |  |  |
| Heart rate, beats/min | 79.6 (71.1-89.5) | 82.3 (73.2-93.9) | <.001 | 82.9 (74.4-91.6) | 86.1 (78.7-97.7) | <.001 |
| Systolic blood pressure, mmHg | 115.4 (106.7-126.5) | 115.9 (106.8-127.7) | .08 | 118.2 (107.9-131.2) | 119.9 (106.5-129.3) | .90 |
| Diastolic blood pressure, mmHg | 58.7 (53.0-65.5) | 59.9 (53.9-66.3) | <.001 | 60.5 (55.5-66.8) | 61.2 (55.4-67.5) | .42 |
| Mean blood pressure, mmHg | 75.2 (69.9- 82.1) | 75.9 (70.6- 83.0) | .005 | 77.9 (70.5- 85.0) | 77.5 (66.9-86.2) | .97 |
| Respiratory rate, beats/min | 18.2 (16.5-20.4) | 18.8 (16.9-21.4) | <.001 | 17.6 (15.7-20.0) | 17.8 (15.8-20.8) | .60 |
| Temperature, $℃$ | 36.8 (36.6-37.0) | 36.9 (36.7-37.2) | <.001 | 36.8 (36.6-37.1) | 36.8 (36.6-37.2) | .56 |
| Oxygen saturation, % | 97.1 (95.7-98.3) | 97.5 (96.1-98.8) | <.001 | 97.3 (95.8-98.4) | 97.5 (96.2-98.7) | .11 |
| **Laboratory results, median (IQR)** |  |  |  |  |  |  |
| Hematocrit, % | 31.5 (28.0-35.6) | 31.9 (27.5-35.9) | .50 | 31.2 (27.7-34.4) | 30.8 (26.9-34.4) | .71 |
| Hemoglobin, g/dL | 10.3 (9.1-11.7) | 10.4 (8.9-11.7) | .22 | 10.3 (9.1-11.4) | 10.1 (8.7-11.3) | .37 |
| Platelet, 10^9^/L | 175.0 (132.3-228.7) | 178.5 (132.7-242.0) | .10 | 187.0 (141.0-234.0) | 176.0 (121.8-248.8) | .57 |
| White blood cell, 10^9^/L | 11.1 (8.4-14.8) | 12.0 (9.1-15.8) | <.001 | 11.7 (9.2-15.2) | 12.2 (9.4-16.5) | .33 |
| Anion gap, mmol/L | 13.0 (11.4-15.5) | 14.3 (12.3-17.0) | <.001 | 10.4 (8.0-12.9) | 11.3 (8.2-14.5) | .05 |
| Blood urea nitrogen, mg/dL | 19.2 (14.0- 29.5) | 24.0 (16.5- 39.0) | <.001 | 19.0 (13.0- 28.0) | 21.3 (14.3-33.5) | .03 |
| Calcium, mg/dL | 8.3 (8.0-8.7) | 8.3 (7.9-8.7) | .63 | 8.2 (7.8-8.5) | 8.2 (7.8-8.7) | .72 |
| Chloride, mmol/L | 104.8 (101.3-107.5) | 105.0 (101.0-108.3) | .17 | 104.9 (102.0-108.0) | 106.0 (102.6-109.6) | .03 |
| Creatinine, mg/dL | 1.0 (0.8-1.4) | 1.1 (0.8-1.7) | <.001 | 1.0 (0.7-1.4) | 1.1 (0.8-1.6) | .008 |
| Glucose, mg/dL | 127.5 (109.0-151.0) | 134.5 (111.0-171.8) | <.001 | 139.2 (117.8-161.3) | 137.5 (118.6-160.5) | .80 |
| Sodium, mmol/L | 138.3 (136.0-140.4) | 139.0 (136.0-142.0) | <.001 | 138.4 (136.0-141.0) | 139.6 (137.0-142.1) | <.001 |
| Potassium, mmol/L | 4.2 (3.9-4.5) | 4.2 (3.9-4.5) | .51 | 4.2 (3.9-4.6) | 4.2 (3.8-4.4) | .53 |
| International normalized ratio | 1.3 (1.2-1.4) | 1.3 (1.2-1.5) | .005 | 1.4 (1.2-1.6) | 1.5 (1.2-1.8) | .02 |
| Prothrombin time, s | 14.1 (12.5-15.4) | 14.3 (12.4-16.1) | .005 | 16.0 (14.2-18.3) | 17.0 (14.2-20.2) | .02 |
| partial thromboplastin time, s | 31.3 (27.8-38.2) | 31.6 (27.5-39.0) | .92 | 35.5 (35.4-35.6) | 35.4 (34.5-35.6) | .01 |
| Urine output, ml | 1470.0 (975.0-2150.0) | 1230.0 (750.0-1845.0) | <.001 | 1330.0 (874.7-1775.0) | 1074.5 (630.5-1862.5) | .04 |
| **Comorbidity, n (%)** |  |  |  |  |  |  |
| Hypertension | 3496.0 (78.2) | 1341.0 (80.9) | .02 | 104.0 (18.0) | 26.0 (19.7) | .65 |
| Diabetes | 1455.0 (32.5) | 621.0 (37.5) | <.001 | 78.0 (13.5) | 18.0 (13.6) | .97 |
| Congestive heart failure | 1525.0 (34.1) | 659.0 (39.7) | <.001 | 50.0 (8.7) | 6.0 (4.5) | .11 |
| Chronic renal disease | 1097.0 (24.5) | 521.0 (31.4) | <.001 | 48.0 (8.3) | 10.0 (7.6) | .78 |
| Chronic liver disease | 366.0 (8.2) | 146.0 (8.8) | .44 | 6.0 (1.0) | 4.0 (3.0) | .10 |
| Chronic pulmonary disease | 1174.0 (26.3) | 494.0 (29.8) | .006 | 55.0 (9.5) | 12.0 (9.1) | .88 |
| Peptic ulcer disease | 137.0 (3.1) | 59.0 (3.6) | .33 | 4.0 (0.7) | 1.0 (0.8) | .99 |
| Tumor | 749.0 (16.8) | 237.0 (14.3) | .02 | 122.0 (21.1) | 22.0 (16.7) | .25 |
| Dementia | 116.0 (2.6) | 226.0 (13.6) | <.001 | 7.0 (1.2) | 4.0 (3.0) | .13 |
| **Score, median (IQR)** |  |  |  |  |  |  |
| GCS^d^ | 15.0 (14.0-15.0) | 14.0 (12.0-15.0) | <.001 | 14.0 (11.0-15.0) | 13.0 (7.5-14.0) | <.001 |
| SOFA^e^ | 4.0 (2.0-6.0) | 6.0 (3.0-9.0) | <.001 | 5.0 (4.0-7.0) | 7.0 (5.0-9.0) | <.001 |
| APSIII^f^ | 38.0 (30.0-49.0) | 49.0 (38.0-63.0) | <.001 | 40.0 (30.0-53.0) | 49.5 (36.0-67.0) | <.001 |
| **Treatment measures, n (%)** |  |  |  |  |  |  |
| Renal replacement therapy | 145.0 (3.2) | 85.0 (5.1) | <.001 | 20.0 (3.5) | 4.0 (3.0) | .99 |
| Invasive ventilation | 1794.0 (40.1) | 1091.0 (65.8) | <.001 | 219.0 (38.0) | 62.0 (47.0) | .06 |
| Acetaminophen | 3471.0 (77.6) | 1091.0 (65.8) | <.001 | 287.0 (49.7) | 83.0 (62.9) | .006 |
| Anticholinergics | 1408.0 (31.5) | 506.0 (30.5) | .47 | 36.0 (6.2) | 19.0 (14.4) | .002 |
| Anticoagulants | 2906.0 (65.0) | 1180.0 (71.2) | <.001 | 210.0 (36.4) | 46.0 (34.8) | .74 |
| Antihistamines | 237.0 (5.3) | 67.0 (4.0) | .04 | 37.0 (6.4) | 5.0 (3.8) | .25 |
| Antipsychotics | 173.0 (3.9) | 188.0 (11.3) | <.001 | 7.0 (1.2) | 4.0 (3.0) | .13 |
| Benzodiazepines | 691.0 (15.5) | 249.0 (15.0) | .67 | 94.0 (16.3) | 29.0 (22.0) | .12 |
| Diuretics | 1822.0 (40.8) | 630.0 (38.0) | .05 | 139.0 (24.1) | 33.0 (25.0) | .83 |
| General anesthetics | 1654.0 (37.0) | 943.0 (56.9) | <.001 | 74.0 (12.8) | 26.0 (19.7) | .04 |
| NSAIDs^g^ | 2333.0 (52.2) | 618.0 (37.3) | <.001 | 114.0 (19.8) | 34.0 (25.8) | .13 |
| Opioids | 3840.0 (85.9) | 1477.0 (89.1) | <..001 | 294.0 (51.0) | 81.0 (61.4) | .03 |
| Vasopressors | 2199.0 (49.2) | 892.0 (53.8) | <..001 | 97.0 (16.8) | 38.0 (28.8) | .002 |

^a^MIMIC-IV: Medical Information Marketplace for Intensive Care IV.

^b^eICU-CRD: eICU Collaborative Research Database.

^c^ICU: intensive care unit.

^d^GCS: Glasgow Coma Scale.

^e^SOFA: Sequential Organ Failure Assessment.

^f^APSIII: Acute Physiology Score III.

^g^NSAIDs: Nonsteroidal Antiinflammatory Drugs.
