# Supplementary material for: Development of a Machine Learning–Based Predictive Model for Postoperative Delirium in Older Adult Intensive Care Unit Patients: Retrospective Study
Source: J Med Internet Res. 2025 Jun 19;27:e67258. doi: 10.2196/67258 (PMC12226778; doi:10.2196/67258)

Multimedia Appendix 5: Confusion Matrix of XGB Models for Different Prediction windows in the external validation set.


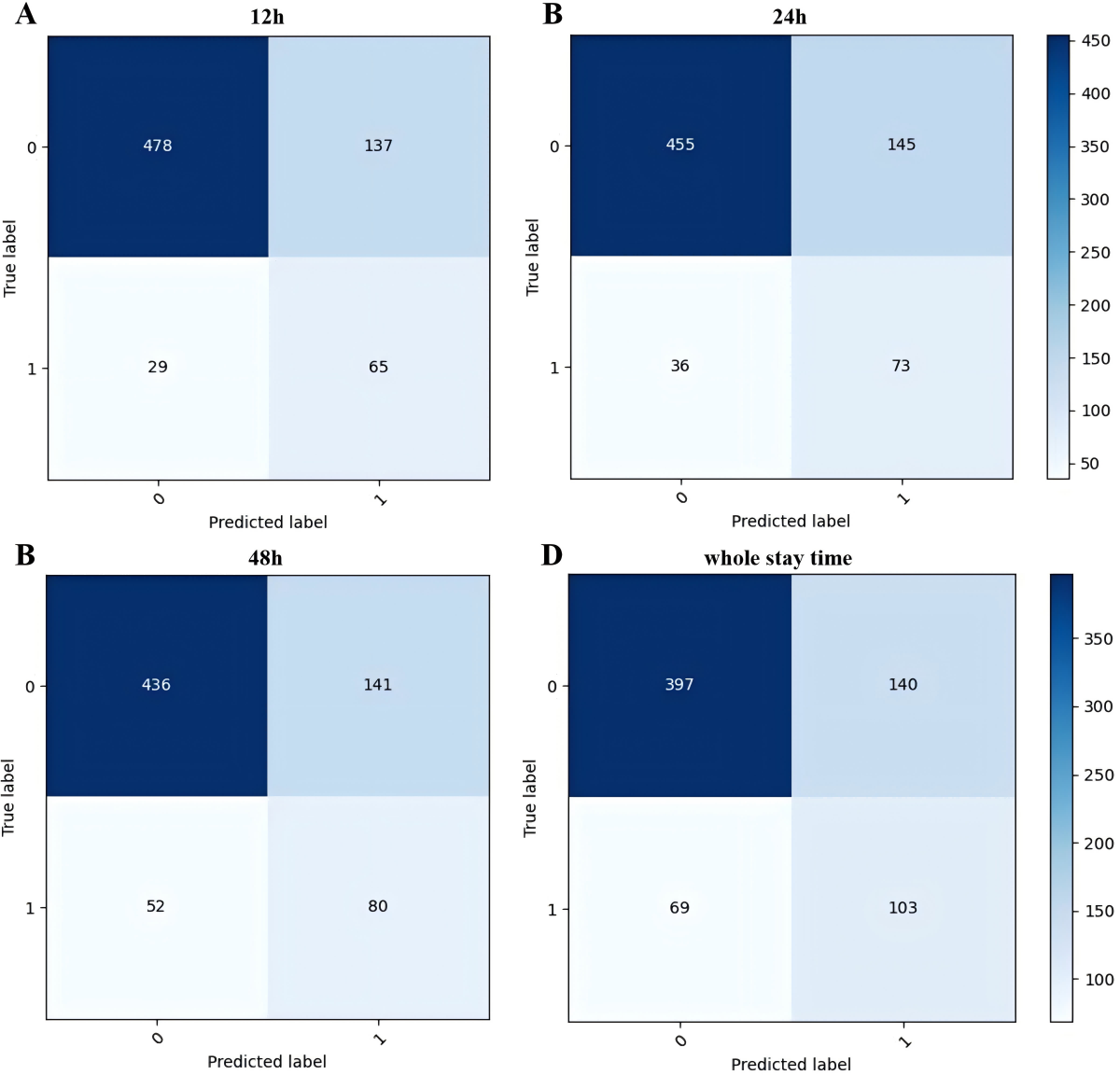

Supplement: Multimedia Appendix 5 [file jmir_v27i1e67258_app5.docx]
